# Evaluating Spin–Orbit Effects on the Thermochemistry of Proton-Coupled Electron Transfer

**DOI:** 10.1021/acs.inorgchem.5c05144

**Published:** 2025-12-22

**Authors:** Daniel Delony, Arnd Fitterer, Martin Diefenbach, Florian Wätjen, Sandipan Maji, Serhiy Demeshko, Matthias Otte, Milan Orlita, Vera Krewald, Max C. Holthausen, Sven Schneider

**Affiliations:** † 9375 Universität Göttingen, Institut für Anorganische Chemie, Tammannstraße 4, Göttingen 37077, Germany; ‡ 9173 Goethe-Universität Frankfurt, Institut für Anorganische Und Analytische Chemie, Max-von-Laue Str. 7, Frankfurt Am Main 60438, Germany; § TU Darmstadt, Department of Chemistry, Quantum Chemistry, Peter-Grünberg-Straße 4, Darmstadt 64287, Germany; ∥ 129851 Laboratoire National des Champs Magnetiques Intenses, 25 Rue des Martyrs, Grenoble 38042, France; ¶ Institute of Physics, Charles University, Ke Karlovu 5, Prague 121 16, Czech Republic

## Abstract

Many heavy transition metal compounds are active redox catalysts. Their redox potentials can be offset by differential spin–orbit coupling (SOC) effects in the case of strong perturbation of the ground-state energy of the oxidized *or* the reduced state. However, SOC effects are often considered negligible in the case of organometallic species, anticipating energetically well-separated, nondegenerate spin ground states for metal ions in strong ligand fields with low symmetry. We here report a rhenium­(III) aminodiphosphine complex that undergoes proton-coupled electron transfer with a phenoxyl radical as a hydrogen abstractor. Experimental derivation of the PCET thermochemistry shows a deviation from coupled-cluster computations in the range of 6 kcal·mol^–1^. The deviation can be attributed to a sizable SOC contribution by the amine precursor, which is largely quenched in the rhenium­(IV) amido product. Our case study emphasizes potential pitfalls for coupled-cluster benchmarking of the reaction energetics of heavy d-block catalysts.

## Graphical abstract

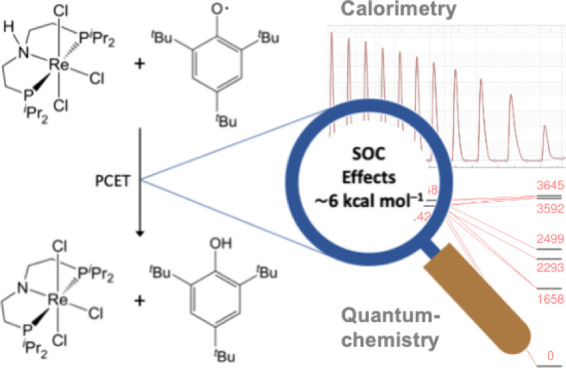

## Introduction

Redox reactions are ubiquitous in biology, energy conversion, and chemical synthesis. Heavy metal catalysts often exhibit intriguing performance with prominent examples spanning commercial water electrolysis (Pt and IrO_2_), W-catalyzed molecular N_2_ electroreduction, or the recent emergence of bismuth catalysis.^1–3^ In many—if not most—cases, quantum chemical methods are now routinely applied to elucidate the pertinent electronic structures and reaction mechanisms, which thus necessitate an accurate treatment of relativistic effects.^4^ For instance, Koch and coworkers demonstrated that the thermochemistry of methane activation by bare Pt ions in the gas phase is governed by scalar relativistic effects and spin–orbit coupling (SOC).^5^

Scalar relativistic effects are routinely accounted for in quantum chemistry by using relativistic effective core potentials.^6^ In contrast, the impact of SOC contributions on solution-phase thermochemistry has been examined much less extensively. As a rare reference study, Srnec et al. examined the redox potentials of heavy group 8 complexes by means of multireference computations with perturbational treatment of SOC.^7,8^ The authors reported sizable SOC contributions of up to Δ*E*_SOC_ ≈ −390 mV (=–9.0 kcal·mol^–1^) for compounds with idealized *O*_h_ symmetry, such as [Os­(H_2_O)_6_]^3+/2+^. These effects were attributed to SOC-induced splitting of the degenerate ^2^T_2g_(Os^III^) spin ground state into spin–orbit states, whereas the nondegenerate ^1^A_1g_ state of the Os^II^ ion is hardly affected by SOC. In contrast, much smaller SOC contributions were computed for the [Ru­(H_2_O)_6_]^3+/2+^ redox couple (Δ*E*_SOC_ ≈ −120 mV). Thus, differential SOC contributions of chemical relevance (>1–2 kcal·mol^–1^) can be expected in cases where only one of the two redox states exhibits a degenerate spin ground state.

Strong ligand fields with low symmetry, in turn, may sufficiently isolate the spin ground state and render SOC contributions to the redox potential chemically insignificant. Indeed, SOC effects on the thermochemistry of organometallic reactions are often negligible.^9–12^ For example, we examined proton-coupled electron transfer (PCET) reactions of the hydroxo/oxo couple [Ir^II^(OH)­{N­(CHCHP^*t*^Bu_2_)_2_}] and [Ir^III^(O)­{N­(CHCHP^*t*^Bu_2_)_2_}] (Scheme 1A).^13,14^ The square-planar iridium compounds exhibit energetically well-separated ^2^ *A* and ^3^ *A* ground states, respectively, and therefore a negligible SOC contribution to the PCET thermochemistry. In contrast, the H atom transfer reaction in Scheme 1B appears to be driven by a sizable SOC effect arising from additive contributions of the Ir–H/Ir and the {Re^IV^(NBz)}/{Re^III^(NHBz)} PCET couples (Δ*E*_SOC_ = −8.7 kcal·mol^–1^ and −5.8 kcal·mol^–1^, respectively).^15^ This computational result is somewhat surprising given the low symmetry of the Re complex. The transient nature of the starting states, however, prevented experimental validation. We therefore set out to conduct a benchmarking study with a related system of low molecular symmetry (*C*_S_) that undergoes clean H atom transfer with a suitable acceptor, such as the 2,4,6-tris-*tert*-butylphenoxyl radical (Mes*O; Scheme 2). Charge-neutral PCET was also chosen^16,17^ because its driving force is less sensitive to solvation than that of pure ET reactions, allowing a more robust computational treatment.

**Scheme 1:**
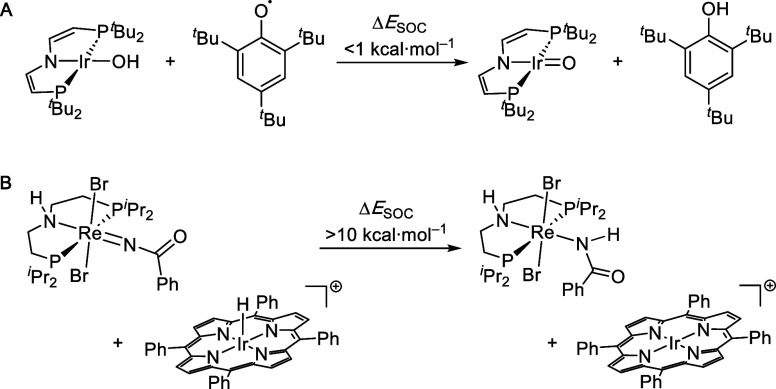
Examples for Low (**A**) and High (**B**) SOC Contributions to PCET Thermochemistry Reported by Our Group^13,15^

**Scheme 2:**
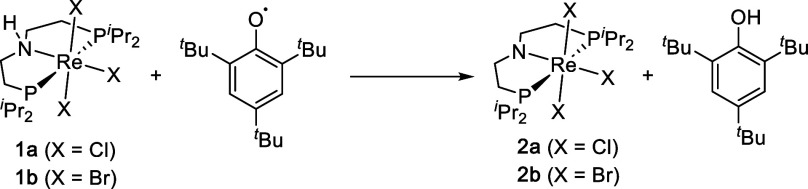
PCET Reaction Examined in this Study

The goal of this case study is to quantify SOC effects on the reaction thermochemistry by comparing isothermal titration calorimetry (ITC) data to high-level quantum-chemical results. For the full molecular systems, we employed DLPNO–CCSD­(T_1_) theory as the most rigorous method feasible at this scale. Refined energies were obtained through an ONIOM single-point extrapolation incorporating explicitly correlated CCSD­(T*)-F12 results for the truncated Re­(PNP) core and unsubstituted phenol. These calculations, denoted CC^ONIOM^ in the following text, include only spin-free relativistic effects via an effective core potential for Re. SOC contributions are not captured in this treatment but were evaluated independently using multiconfigurational CASSCF/NEVPT2/QDPT theory.

In the following, we first assess the ground-state electronic structures of PCET couples **1a** and **2a** as a computational benchmark. We then compare the experimentally and quantum-chemically derived PCET driving forces to isolate and quantify the SOC contribution to the reaction thermochemistry.

## Results and Discussion

### Spectroscopic Characterization of the Precursors

We recently reported the synthesis of the rhenium­(III) complexes [ReX_3_(^H^PNP)] (X = Cl (**1a**), Br (**1b**); ^H^PNP = HN­(CH_2_CH_2_P^*i*^Pr_2_)_2_; Scheme 2) as precursors for reductive N_2_ splitting.^18,19^ The paramagnetically shifted (δ_31P_ = −1526 (**1a**), 1488 (**1b**) ppm) yet sharp NMR signals are typical for high-spin rhenium­(III) due to rapid electronic relaxation.^20,21^ The chemical shifts exhibit little temperature dependence except for the N–H protons, presumably due to hydrogen bonding with the solvent. All others approximately scale linearly with temperature (δ ∝ *T*, Figure S12), as was previously reported for Re^III^ complexes.^22–24^ While Curie behavior of the isotropic shielding (δ ∝ *T* ^–1^) is expected for *S* = 1/2 systems,^25^ higher inverse order temperature dependence can arise for higher spin states with large axial zero-field splitting (*D*) and *g*-anisotropy.^26^ The small and linear temperature dependence observed for **1a**/**b** supports a thermally isolated (Δ*E* ≫ *k*_B_ *T*), nonmagnetic ground state with excited state admixture in the magnetic field that leads to second-order (Van Vleck) paramagnetism.

This electronic structure picture from NMR is confirmed by magnetic measurements in the solid state (Figures 1a and S13). The room-temperature magnetic moments of **1a**/**b** $\left(\mu_{\mathrm{eff}}^{298K}=1.60\mu_{B}\right)$ are significantly smaller than the *S* = 1 spin-only values and are in the typical range for octahedral Re^III^ complexes. Temperature-independent magnetic susceptibility (TIP; χ_M_/cm^3^·mol^–1^ = 1063 × 10^–6^ (**1a**), 1083 × 10^–6^ (**1b**)) is observed over the full range and is typical for octahedral 5d^4^ complexes (Re^III^, Os^IV^).^21,27–30^

**Figure 1:**
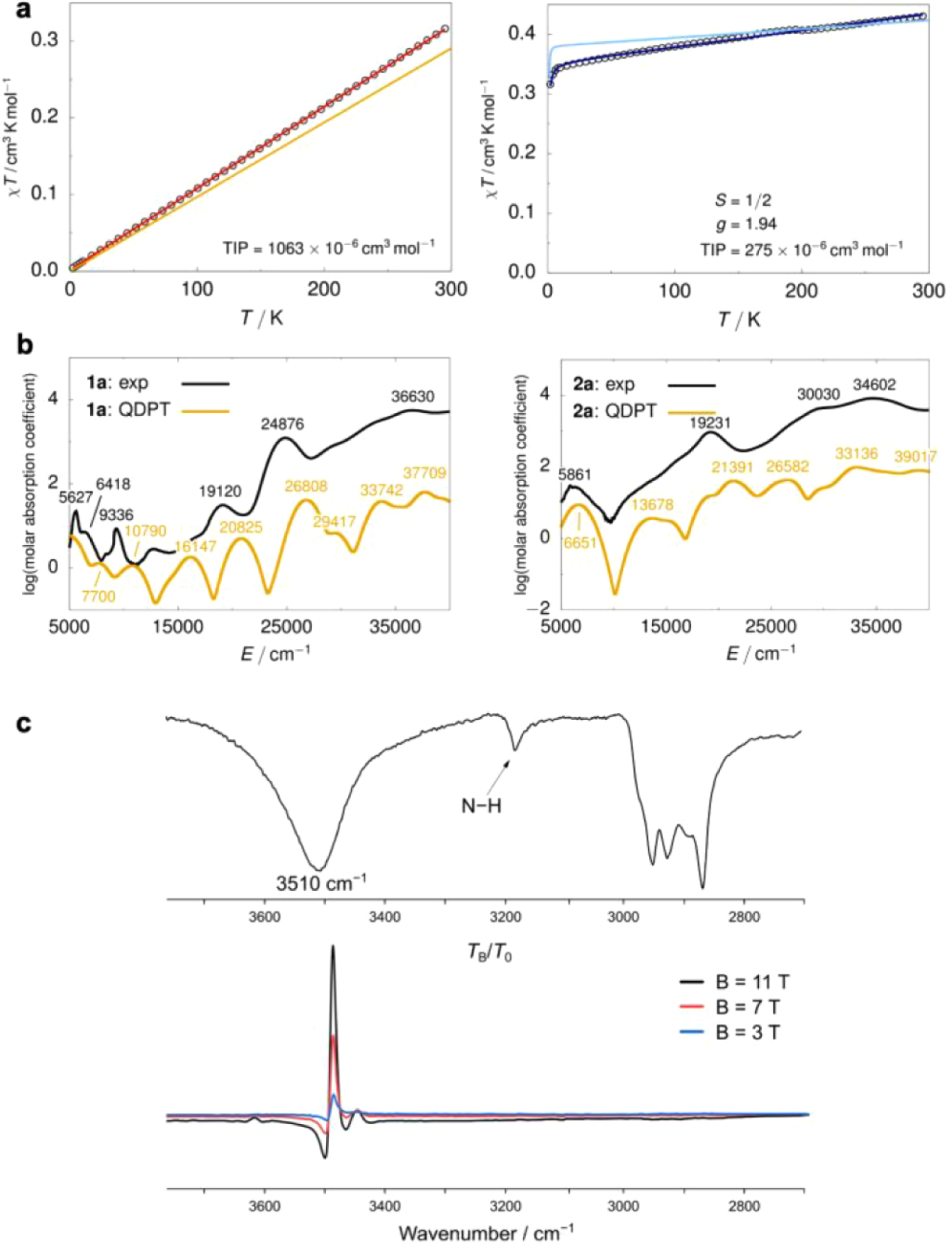
Magnetic and spectroscopic data. (a) SQUID magnetometry data of **1a** (left) and **2a** (right) at 0.5 T: experimental data (black circles), simulation data (red/blue lines with simulation parameters), and *ab initio* data (orange/turquoise lines). (b) UV–vis/NIR spectra of **1a** (left) and **2a** (right): experimental data in THF (black) and *ab initio* computed data (yellow, Gaussian line broadening set to fwhm = 1500 cm^–1^, *y*-values scaled by 0.2). (c) Expansion of the IR spectrum of **1a** (top) and field-dependent IR spectra (bottom) at 4.2 K is shown as transmission spectra at applied field (*T*_B_) divided by the zero-field spectrum (*T*_0_).

The electronic absorption spectra show dominating charge-transfer bands around 25,000 cm^–1^ (**1a**: ε = 1.2 × 10^3^ M^–1^cm^–1^; Figure 1b). In addition, several weak NIR bands (ε < 50 M^–1^cm^–1^) were found, which extend all the way to the mid-IR (3510 (**1a**) and 3462 (**1b**) cm^–1^), next to the sharper C–H (∼2900 cm^–1^) and N–H (∼3200 cm^–1^) vibrational transitions (Figure 1c).^31^ Ligand-field bands in the IR range were previously reported for *mer*-Re^III^X_3_L_3_ trisphosphine complexes.^32,33^ For **1a**, this assignment was confirmed by magnetic IR spectroscopy, exploiting the magnetic field dependence of states with |*M*_J_| > 0. The feature at ∼3500 cm^–1^ exhibits a rising intensity in the normalized transmission plots (*T*_B_/*T*_0_), confirming its origin from an electronic transition.

We previously reported that the rhenium­(IV) amido complex [Re^IV^Cl_3_(PNP)] (**2a**) results from PCET of **1a** with Mes*O as a hydrogen atom acceptor (Scheme 2).^18^ In analogy, the bromo complex **2b** is obtained from **1b** and Mes*O in 63% isolated yield. The room-temperature magnetic moments of **2a** and **2b** ( $\mu_{\mathrm{eff}}^{298K}$ = 1.86 μ_B_ and 1.84 μ_B_, respectively) are larger than those of the parent **1a**/**b** and relatively close to the spin-only value for a doublet ground state (1.73 μ_B_). Accordingly, the temperature-dependent magnetic data (Figures 1a and S14) could be fitted for low-spin (*S* = 1/2) ground states (*g*_av_ = 1.94 (**2a**), 2.00 (**2b**)) with pronounced, yet much smaller TIP (χ_M_/cm^3^·mol^–1^ = 275 × 10^–6^ (**2a**), 870 × 10^–6^ (**2b**)) compared to **1a**/**b**. This finding suggests a smaller perturbation of the spin ground state by SOC. Spectroscopic characterization also showed electronic transitions in the mid-IR range at significantly lower energy (1967 (**2a**) and 1861 (**2b**) cm^–1^).

### Electronic Structure Calculations

CC^ONIOM^ calculations on **1a** establish an electronic triplet ground state with a sizable adiabatic gap to the lowest singlet state (Δ*E*_S–T_ = 4.2 kcal·mol^–1^). State-averaged CASSCF­(14,10)/NEVPT2 calculations uncover an accidental near-degeneracy of the two lowest lying d orbitals, d_*xy*_ and d_*yz*_, which together host 3 electrons. Consequently, the multireference picture produces the two lowest triplet states that are separated by merely 124 cm^–1^, each dominated by linear combinations of the (d_*xy*_)^2^(d_*yz*_)^1^(d_*xz*_)^1^ and (d_*xy*_)^1^(d_*yz*_)^2^(d_*xz*_)^1^ configurations (see Supporting Information for further detail).

The accidental quasi-degeneracy of the two lowest triplet states represents a “quasi-^3^ *E*” spin ground state, even in a low symmetry field. Thus, sizable SOC effects are to be expected.^7^ The ground state can be derived from the parent Re^III^ in an octahedral ligand field (*O*_h_). Its ^3^ *T*_1g_ spin ground state is expected to split by large SOC (*z*_Re(III)_ = −2500 cm^–1^) to give an isolated *J* = 0 spin–orbit ground state.^34^ The higher spin–orbit levels are further split in lower symmetry. For example, tetragonal distortion (*C*_4_) gives rise to six microstates that originate in ^3^ *E* (*e* ^3^ *b* ^1^) and ^3^ *A* (*b* ^2^ *e* ^2^) orbital states, respectively.^30^ In the case of **1a** (*C*_s_), SOC effects evaluated by CASSCF­(14,10)/NEVPT2/QDPT result in a large splitting of the multireference eigenstates (Figure 2). The spin–orbit ground state is composed of the two lowest triplet states and is strongly stabilized with respect to the NEVPT2 states (Δ*E*_SOC_ = −3424 cm^–1^ = −9.8 kcal·mol^–1^; Table S17). The influence of ligand field distortions on Δ*E*_SOC_ was probed by symmetrically displacing the axial halide ligands of **1a** and **2a** by ±0.05 Å along the Re–Cl bonds. Δ*E*_SOC_ varies only marginally for **1a** and slightly more for **2a** (Table S21). However, the magnitude of ΔΔ*E*_SOC_ remains within a rather narrow range (4.2–6.7 kcal·mol^–1^), indicating that the spin-state near-degeneracy is lifted predominantly by SOC rather than by Jahn–Teller distortion.

**Figure 2:**
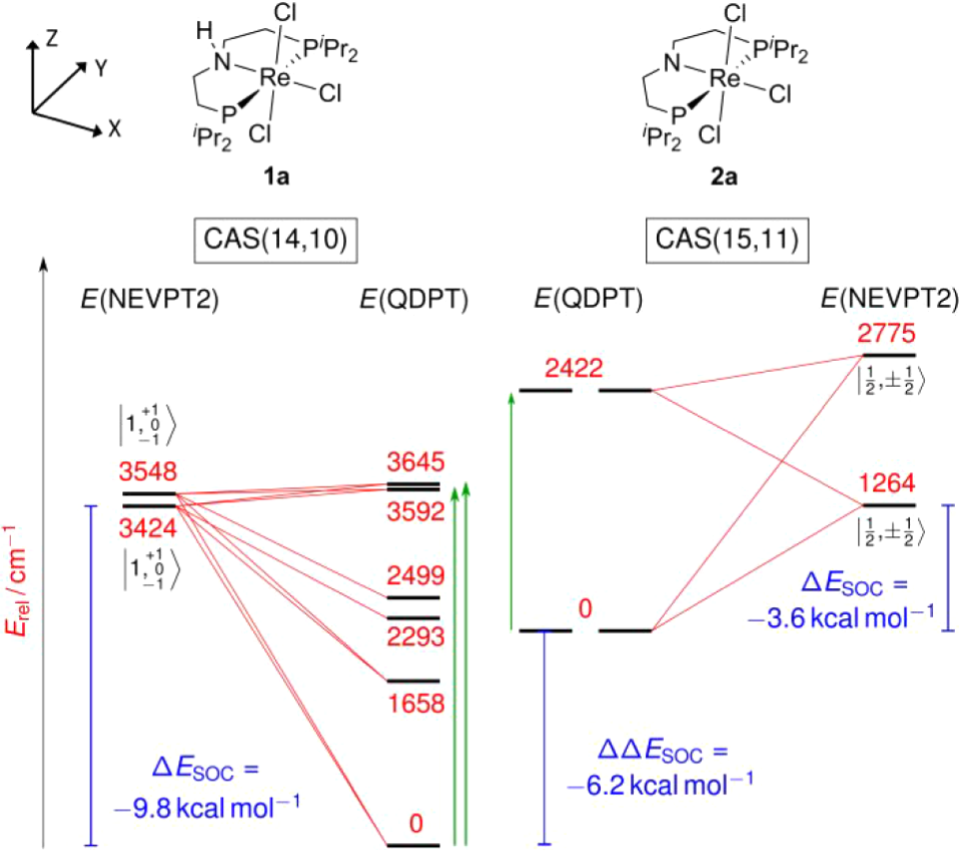
CASSCF/NEVPT2/QDPT state correlation diagram for the lowest-lying states of **1a** (*left*) and **2a** (*right*). Spin–orbit coupling (SOC) stabilization energies are indicated with blue bars, and electronic excitations with significant oscillator strength are indicated with green arrows. The larger SOC-induced splitting of the almost degenerate triplet ground state of **1a** affords additional stabilization relative to the less SOC-stabilized doublet ground state of **2a**, thereby offsetting the driving force for the PCET process.

CC^ONIOM^ calculations on the rhenium­(IV) complex **2a** establish a metal-centered doublet ground state that lies well below the quartet state (Δ*E*_D–Q_ = −8.3 kcal·mol^–1^). This stabilization arises from strong π-donation by the amido ligand, reflected in the pronounced shortening of the Re–N bond compared to that in **1a** (Δ*d*_PBE0_ = 0.28 Å; Δ*d*_exp_ = 0.26 Å).^18^ Consequently, the d_*xz*_ orbital is increased in energy, thereby strongly favoring a low-spin configuration. CASSCF­(15,11)/NEVPT2 calculations corroborate a doublet ground state dominated by the (d_*xy*_)^2^(d_*yz*_)^1^ configuration (82%). The first excited doublet state lies considerably higher in energy, at 1511 cm^–1^ (Figure 2).^35^ QDPT evaluation of SOC-induced state mixing reveals a ground-state stabilization of Δ*E*_SOC_ = −1264 cm^–1^ (−3.6 kcal·mol^–1^), which is notably smaller than for **1a**.

The CASSCF/NEVPT2/QDPT computations reproduce the experimental magnetic and spectroscopic data for **1a** and **2a** reasonably well (Figure 1 and Table 1). Notably, the quantum chemical analysis suggests that the experimental IR feature at 3510 cm^–1^ provides, albeit fortuitously, a measure of Δ*E*_SOC_ for **1a**, which we can directly relate to the computed value of 3592 cm^–1^ (2% deviation).

**Table 1:** Spectroscopic and Magnetic Data of **1a** and **2a**: Comparison of Experimental and CASSCF/NEVPT2/QDPT Computed Data

	1a		2a	
	Experiment^a^	QDPT^b^	Experiment^a^	QDPT^b^
μ_eff,RT_ (μ_B_)	1.60	1.52	1.86	1.84
UV/vis (cm^–1^)	24876 (1238)	26808	30030 (4446)	33136
	19120 (31)	20825	19231 (934)	21391
NIR (cm^–1^)	9336 (9)	10790		
	6418 (7)	7700	5861 (30)	6651
	5627 (22)	5063		
IR (cm^–1^)	3510 (n.d.)	3592	1967 (n.d.)	2422

a ε in M^–1^ cm^–1^ in parentheses.

b Selected transitions with significant f_osc_.

### PCET Thermochemistry: Experimental Determination

The electronic structure characterization indicates a sizable differential SOC contribution to hydrogen abstraction from **1** of around Δ*E*_SOC_ = −6.2 kcal·mol^–1^ (Scheme 2). This magnitude can be relevant for chemical reactivity and is well beyond the error margins of solution-phase thermochemical methods. For benchmarking, two independent approaches were pursued to derive the reaction energetics with the reference hydrogen acceptor Mes^∗^O (Scheme 2; *BDFE*_O–H_(Mes∗OH) = 74.4 kcal·mol^–1^ in THF).^17^

On one hand, the N–H bond dissociation free energy of **1** (*BDFE*_N–H_) can be derived via Bordwell’s equation (eq 1), which refers to a Hess cycle (“square scheme”) that breaks down the formal driving force for N–H dissociation into electron and proton transfer contributions (*E* ^0^, p*K*_a_), as well as the solvent-dependent free energy of the hydrogen atom (*C*_G_).^16^

1 $$\mathrm{BDFE}=23.06\cdot E^{0}+1.37\cdot pK_{a}+C_{G}$$

From there, the free reaction energy for H-transfer to Mes*O (Δ_r_ *G* ^0^) is easily calculated for comparison with computations. On the other hand, the selective reaction of **1** with Mes*O directly enables thermochemical analysis by isothermal titration calorimetry (ITC).^13,36,37^ Note that ITC offers reaction enthalpies (Δ_r_ *H* ^0^), thus eliminating the computational uncertainties that are associated with estimating the reaction entropy.

The Re^III^ amine complexes **1a**/**b** exhibit reversible 1e^–^ oxidations in the cyclic voltammogram (THF) at *E* ^0^ = −0.29 (**1a**) and 0.22 (**1b**) ± 0.01 V.^38^ The small anodic shift for the exchange of chloride with bromide (Δ*E* ^0^ = 0.07 V) is in line with Lever’s electrochemical ligand parameters (3·Δ*E*_L_ = 0.06 V)^39^ and is much less pronounced than for the five-coordinate Re^III^ dihalide amido complexes [ReX_2_{N­(CH_2_CH_2_PtBu_2_)_2_}] (X = Cl, Br, I).^40^ The rhenium­(IV) reaction products, [ReX_3_(^H^PNP)]^+^ (X = Cl (**3a**), Br (**3b**)), were prepared by chemical oxidation of **1a**/**b** with AgBAr^F^_24_ (**1a**; BAr^F^_24_^–^ = B­{C_6_H_3_(3,5-CF_3_)_2_}_4_^–^) and [FeCp_2_]­BAr^F^_24_ (**1b**), respectively, in yields around 75%. **3a**/**b** were fully characterized including crystallography (see Supporting Information). p*K*_a_(N–H) determination of **3a**/**b** in THF was carried out by ITC titration with pyridine (**3a**) and lutidine (**3b**) as reference bases, respectively. Clean formation of the amide complexes **2a**/**b** was confirmed spectroscopically by NMR. Using the reported p*K*_a_ values of pyridine (5.5) and lutidine (7.2) in THF,^41^ fitting of the thermogram gave p*K*_a_ values of 5.3 ± 0.1 (**3a**) and 6.3 ± 0.1 (**3b**), respectively. From this data, eq 1 gives *BDFE*_N–H_ values of 60.5 ± 2.4 (**1a**) and 63.5 ± 2.4 (**1b**) kcal·mol^–1^ (Scheme 3), using the free energy of the hydrogen atom in THF recommended by Mayer and coworkers (*C*_G_ = 59.9 kcal·mol^–1^).^17^ Thus, free reaction energies for PCET with Mes*O (Scheme 2) in THF of Δ_r_ *G* ^0^ = −13.9 ± 2.6 (**1a**) and −10.9 ± 2.6 (**1b**) kcal·mol^–1^ are derived.

**Scheme 3:**
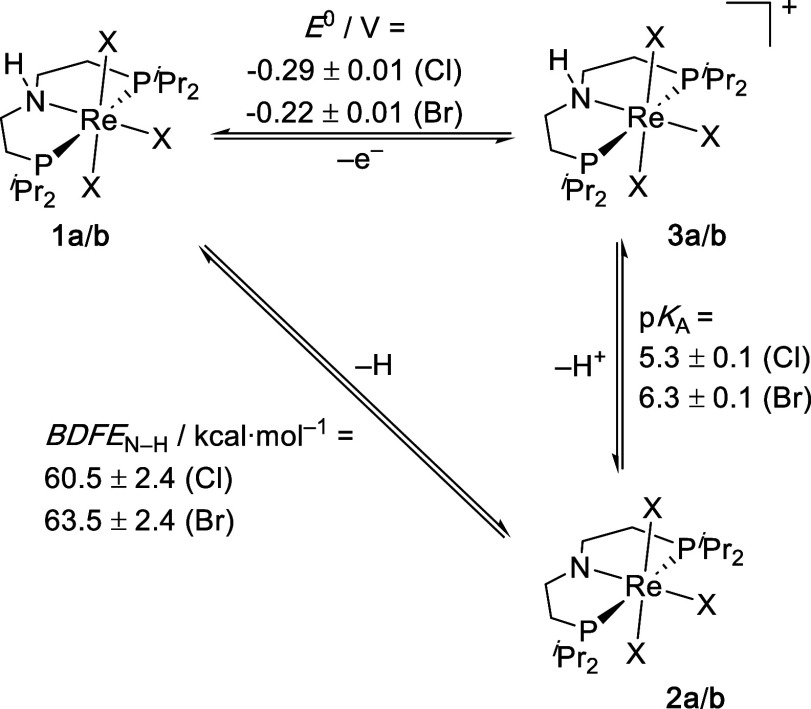
PCET Square Scheme of **1a/b** in THF

In the alternative approach, ITC titration with Mes*O was carried out in the less polar solvent dichloromethane, further reducing the propensity for hydrogen bonding with the solvent. In addition, titration in THF led to significant tailing and baseline shifts in the thermograms, indicating an underlying slower side reaction. Synthetic examination of **1a** confirmed partial overoxidation by Mes*O in THF due to dehydrogenation of the pincer backbone to give the Re^III^ imine complex [ReCl_3_{N­(CHCH_2_P*i*Pr_2_)­(CH_2_CH_2_P*i*Pr_2_)}] as a side product.^18^ Unfortunately, tailing of the thermogram trace was also observed for the titration of **1b** in dichloromethane. ITC results for **1b** were therefore discarded. Titration of **1a** in CH_2_Cl_2_ revealed a reaction enthalpy of Δ_r_ *H* ^0^ = −12.2 ± 0.1 kcal·mol^–1^ (Scheme 4). The exothermic character of the reaction prevented meaningful fitting of the thermogram for the derivation of Δ_r_ *G* ^0^.

**Scheme 4:**
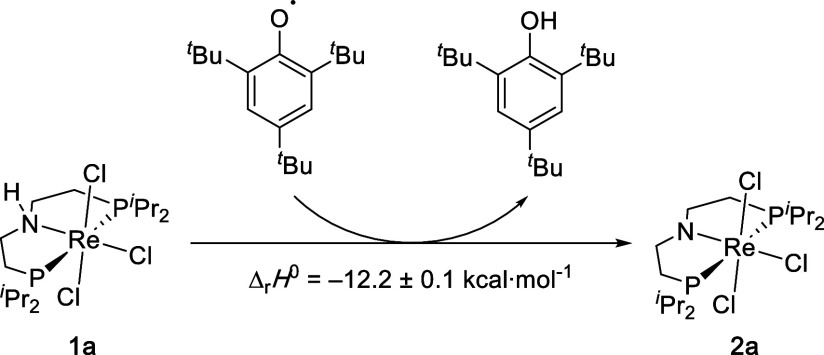
Calorimetric Titration Experiment for the PCET Reaction of **1a** with Mes*O in Dichloromethane

Notably, the experimental values of Δ_r_ *G* ^0^ in THF and Δ_r_ *H* ^0^ in dichloromethane obtained from the two approaches for **1a** are identical within error. This observation is in line with findings from Mayer et al. that (a) entropic contributions to the hydrogen transfer thermochemistry nearly cancel in the absence of major spin changes that affect the vibrational entropy and (b) *BD­(F)­E*s generally exhibit low solvent dependence.^17,42,43^

### PCET Thermochemistry: Quantum-Chemical Evaluation

Based on a H-truncated model system, we performed a series of benchmark calculations to establish the accuracy that can be expected from our computational approach. Comparison of extrapolated CCSD­(T*)-F12/CBS­(DT) results obtained with HF, PBE0, and BP86 reference wave functions for spin-state splittings in **1a** ^**model**^ and **2a** ^**model**^, the PCET reaction energy Δ*E*_r_, and the homolytic dissociation energies Δ*E*_N–H_(**1a** ^**model**^) and Δ*E*_O–H_(phenol) show an overall agreement within 0.8 kcal·mol^–1^ (cf. Table S9). None of the usual coupled-cluster diagnostics indicated any alarming problem; thus, the use of KS reference wave functions provides no obvious advantage. We therefore chose the HF-based CCSD­(T*)-F12/CBS­(DT) data as a reference for further comparison and for use within the ONIOM extrapolation discussed further below. Against this data, DLPNO–CCSD­(T_1_)/CBS­(DT)/CPS­(56) results show non-negligible deviations between 0.7 and 2.1 kcal·mol^–1^ for the spin-state splittings and the O–H dissociation energies, while the reaction energy benefits from error compensation. Notably, neither improvement of the extrapolated basis sets, nor of the PNO thresholds, nor the use of KS reference wave functions leads to consistently better agreement with the CCSD­(T*)-F12/CBS­(DT) reference data (Table S9). For improved results, we thus chose the ONIOM approach, which we tested favorably for spin-state splittings (cf. Section S6.3 in the Supporting Information).

After inclusion of thermochemical contributions from the DFT Hessian analyses to the CC^ONIOM^ results, we obtain a reaction enthalpy of Δ*H*_r_ = −18.4 kcal·mol^–1^ and upon inclusion of SOC effects, Δ*H*_r_^SOC^ = −12.2 kcal·mol^–1^, matching perfectly the experimental value (−12.2 ± 0.1 kcal·mol^–1^). With Δ*H*_r_ = −18.0 kcal·mol^–1^ and Δ*H*_r_^SOC^ = −11.8 kcal·mol^–1^, the DLPNO–CCSD­(T_1_)/CBS­(DT)/CPS­(56) results also show pleasing agreement, demonstrating consistency of the chosen computational protocols. Our results illustrate the chemical significance of the SOC effect (ΔΔ*E*_SOC_ = −6.2 kcal·mol^–1^), which contributes 51% to the overall PCET driving force.

## Conclusions

This combined experimental and computational benchmark study demonstrates a sizable SOC offset to the PCET thermochemistry of our heavy d-block model complex by more than 6 kcal·mol^–1^. The large differential relativistic effect on the PCET thermochemistry can be ascribed to the accidental near-degeneracy of the two lowest spin configurations of amine complex **1a**, which is lifted by SOC. In turn, the rhenium­(IV) PCET product **2a** exhibits an energetically isolated spin ground state due to the strong π-donation of the amido ligand. The spin–orbit ligand field transitions of **1a** and **2a** in the IR spectrum can serve as spectroscopic indicators and reliable benchmark data for computational treatment. ONIOM-based coupled-cluster and DLPNO–CC approaches in combination with CASSCF/NEVPT2/QDPT theory were calorimetrically validated as reliable protocols for computational treatment with high accuracy. Notably, despite the low molecular symmetry of **1a** and **2a** (*C*_S_), the SOC contribution to PCET is remarkably close to that predicted by Srnec et al. for the redox potentials of *O*_h_ symmetric 5d complexes (Δ*E*_SOC_ ≈ −390 mV = −9.0 kcal·mol^–1^).^7^ Our study, thus, emphasizes that chemically significant SOC effects on the thermochemistry of heavy metal ions can be preserved in strong ligand fields with low symmetry and need to be considered for accurate computational treatment of redox reactions.

## Experimental Section

### Experimental Procedures

All experiments were carried out using standard Schlenk or glovebox techniques under an argon atmosphere. Solvents were dried by passing them through columns packed with activated alumina. In addition, THF, benzene, toluene, and pentane were dried over Na/K alloy, distilled by trap-to-trap transfer *in vacuo*, and degassed by three freeze–pump–thaw cycles. 2,4,6-Tris­(*tert*-butyl)­phenoxyl radical (Mes^∗^O), [ReCl_3_(^H^PNP)] (**1a**), and [ReBr_3_(^H^PNP)] (**1b**) were synthesized according to published procedures.^18,19,44^ AgBAr^F^_24_ and [FeCp_2_]­BAr^F^_24_ were purchased from Merck and used without further purification.

Magneto FIR experiments were conducted in the transmission configuration, using the Faraday geometry, where the Poynting vector is parallel to the static magnetic field (**P**||**B**_o_). **1a** was ground in a mortar with eicosane in an approximate ratio of 1:8 and then pressed into pellets. These pellets were placed in a superconducting magnet and kept at *T* = 4.2 K in exchange helium. Then, THz/infrared radiation from a globar was analyzed by a Vertex 80 V FTIR spectrometer, delivered using light-pipe optics to the pellet, and detected by a composite bolometer placed just beneath the pellet. Test spectra were recorded, and the pellets were diluted until the strongest absorptions in the 10–100 cm^–1^ region were between 1 and 5% transmittance. Variable-field spectra between 0 and 16 T were then recorded at the optimal pellet concentration. Background corrections were applied by recording blank reference spectra at the same magnetic fields and taking the ratio of the sample to the reference spectrum at each field. In variable-field maps, the background-corrected transmission spectra were plotted in the form of relative magneto transmission using a differential averaging method developed for emphasizing the field-induced spectral features. The treatment was carried out using the custom-made tool FieldOptic.^45^ All other IR spectra were recorded with a Bruker Alpha Platinum ATR-IR spectrometer. UV/vis NIR measurements and NMR spectra were measured with a Bruker Avance III HD 400 spectrometer.

Isothermal titration calorimetry was performed with a TA Instruments NanoITC calorimeter equipped with a 24K gold cell with a sample volume of 1 mL, operated in overfill mode, and controlled by the ITCRun software version 3.4.6.0. The obtained data were evaluated by the implemented NanoAnalyze software.

Temperature-dependent magnetic susceptibility measurements were carried out with a Quantum Design MPMS-XL-5 SQUID magnetometer equipped with a 5 T magnet in the range from 295 to 2.0 K at a magnetic field of 0.5 T. The freshly isolated crystalline samples were contained in a PTFE bucket and fixed in a nonmagnetic sample holder. Each raw data file for the measured magnetic moment was corrected for the diamagnetic contribution of the PTFE bucket according to *M*_dia_(bucket) = χ_g_·*m*·*H*, with an experimentally obtained gram susceptibility of the PTFE bucket. The molar susceptibility data were corrected for the diamagnetic contribution according to χ_M_^dia^(sample) = −0.5 M × 10^–6^ cm^3^·mol^–1^.^46^ Paramagnetic impurities (PI) with *S* = 1/2 were included according to χ_calc_ = (1 – PI)·χ + PI·χ_mono_. Experimental χ_M_ *T* vs *T* data were modeled with the julX program.^47^ The following Hamiltonian was used for fitting the experimental data:

2 $$Ĥ={g\mu }_{B}\mathrm{B⃗}\mathrm{S⃗}+D\left({Ŝ}_{z}^{2}-1/3S\left(S+1\right)\right)$$

### Syntheses

#### [ReCl_3_(^D^PNP)] (**d_1_-1**)

[ReCl_3_(P^H^NP)] (**1**) (6.0 mg, 10.0 μmol, 1.0 equiv) is suspended in a DCM/D_2_O mixture (0.4 mL/0.1 mL) and stirred overnight. Afterward, the solvent is removed *in vacuo,* and the spectroscopically clean product [ReCl_3_(P^D^NP)] (**1**-*d*) is measured in CD_2_Cl_2_. The NMR spectra show signals largely identical to those of **1**. The signal corresponding to the NH proton is largely gone (∼2% remaining), and the signals at δ_1H_ = −5.10 and −10.44 ppm exhibit different coupling patterns. ^2^H NMR (46.1 MHz, CD_2_Cl_2_, 25 °C): δ (ppm) = 152.6 (s, ND). IR ~ (cm^–1^) = 3506 (br, electronic absorption), 2363 (*v*_ND_).

#### [ReBr_3_(PNP)] (**2b**)

A solution of Mes*O (14.4 mg, 55.2 μmol, 1.1 equiv) in benzene is slowly added to a suspension of **1b** (30.0 mg, 50.2 μmol, 1.0 equiv) in benzene. Stirring for 1 h at room temperature results in a deep red solution. The solvent is removed *in vacuo*, and the crude product is washed with pentane four times. Extraction with benzene and evaporation of the solvent give the product as a purple solid in 63% yield. ^1^H NMR­(C_6_D_6_): δ (ppm) = 21.06 (br, 2H), 18.53 (12H), 16.22 (12H), −2.41 (br, 3H). Anal. Calc. for C_16_H_36_Br_3_NP_2_Re (729.94): C 26.31, H 4.97, N 1.92; Found: C 26.20, H 4.74, N 1.89. LIFDI: *m*/*z* (%) = 729.9 (100)

#### [ReCl_3_(^H^PNP)]­BAr_F_^24^ (**3a**)

To a suspension of **1a** (50.0 mg, 86.6 μmol, 1.00 equiv) in PhCl (10 mL), a solution of AgBAr^F^_24_ (79.4 μmol, 0.95 equiv) in PhCl (10 mL) is added dropwise. The solution is filtered, and the residue is extracted with a small amount of PhCl. Pentane (20 mL) is added to the solution to precipitate the product. The solution is filtered, and the residue is washed with a small amount of pentane and extracted with a small amount of DCM (∼1 mL). Gas-phase layering with pentane yields the product as deep red crystals suitable for single-crystal analysis (53.3 mg, 63.6 μmol, 76%). ^1^H NMR (DCM-*d*_2_): δ (ppm) = 34.3 (2H), 31.7 (6H), 31.1 (6H), 31.0 (6H), 27.0 (6H), 11.7 (2H), −4.6 (2H), −11.0 (2H), −80.4 (2H), −104.9 (2H), N–H not found. Anal. Calc. for C_48_H_49_BCl_3_F_24_NP_2_Re: C 39.46, H 3.38, N 0.96; Found: C 40.02, H 3.33, N 0.93. ATR-IR: ν­(N–H) = 3212 cm^–1^.

#### [ReBr_3_(^H^PNP)]­BAr_F_^24^ (**3b**)

To a solution of **1b** (30.0 mg, 41.0 μmol, 1.0 equiv) in DCM (5 mL), a solution of [FeCp_2_]­BAr^F^_24_ (41.3 mg, 39.4 μmol, 0.96 equiv) in DCM (5 mL) is added dropwise. After 15 min of stirring, the solvent is removed under vacuum. The residue is washed with pentane (5 × 2 mL) and extracted with ether. Removal of the solvent yields the product as a reddish-purple solid (48.4 mg, 30.3 μmol, 74%). Slow evaporation in a pentane solution gave needle-shaped crystals suitable for XRD. ^1^H NMR (THF-*d*_8_): δ (ppm) = 71.1 (N–H), 39.1 (6H), 35.9 (14H), 34.4 (6H), 16.1 (2H), 7.3 (2H), −13.4 (2H), −79.6 (2H), −89.0 (2H). Anal. Calc. for C_48_H_49_BBr_3_F_24_NP_2_Re: C 36.16, H 3.10, N 0.88; Found: C 36.67, H 3.30, N 0.91

## Computational Methods

### DFT Calculations

Using the Gaussian 16 program,^48^ geometry optimizations on the full molecular models were performed employing the PBE0 hybrid density functional^49^ in combination with the D3 dispersion correction^50^ with Becke–Johnson damping^51^ and the def2-TZVP orbital basis set,^52^ including a quasi-relativistic 60-electron pseudopotential for rhenium.^53^ Zero-point vibrational energies, thermal and entropy contributions to obtain enthalpies, and Gibbs free energies were obtained from Hessian analyses as implemented in Gaussian 16.

### DLPNO–CCSD­(T_1_) Calculations.

Based on DFT-optimized geometries, local coupled-cluster single-point energies were computed with the ORCA 6.0.1 program,^54–56^ using the domain-based local-pair natural orbital (DLPNO) approach^57,58^ with improved iterative triples,^59^ DLPNO–CCSD­(T_1_). *VerytightSCF* settings and *tightPNO* default settings were applied without *fullLMP2* guess to ensure consistency for closed-shell and open-shell calculations. Following Altun et al.,^60^ two-point extrapolation to the complete pair natural orbital space (CPS) limit was performed using the Schwenke-style relation^61^

3 $$E^{\infty }=E^{X}+F\left(E^{Y}-E^{X}\right)$$

where *Y* = *X* + 1 and, in the CPS extrapolation context, *E* ^*X*^ and *E* ^*Y*^ are the correlation energies obtained with the corresponding *T*_CutPNO_ thresholds 10^–*X*^ and 10^–*Y*^ and *F* = 1.5 for both CPS(56) and CPS(67) extrapolations. The same ansatz was used for two-point extrapolations to the complete basis set (CBS) limit separately for the reference energy and the correlation energy, employing the def2-SVP (*X* = 2), def2-TZVPP (*X* = 3), and def2-QZVPP (*X* = 4) basis sets (the cardinal numbers are referred to below as D, T, and Q).^52^ For extrapolation of the reference energy, an exponential functional form of the type

4 $$E^{X}=E^{\mathrm{CBS}}+A\cdot e^{\alpha \sqrt{X}}$$

was assumed as originally proposed by Karton and Martin.^62^ This results in the following expression for the two-point CBS extrapolated energy,^60^

5 $$E_{\mathrm{ref}}^{\mathrm{CBS}}=\frac{e^{\alpha \sqrt{Y}}\cdot E_{\mathrm{ref}}^{Y}-e^{\alpha \sqrt{X}}\cdot E_{\mathrm{ref}}^{X}}{e^{\alpha \sqrt{Y}}-e^{\alpha \sqrt{X}}}$$

or, equivalently, in eq 3 with a factor *F* of

6 $$F_{\mathrm{ref}}=\frac{e^{\alpha \sqrt{Y}}}{e^{\alpha \sqrt{Y}}-e^{\alpha \sqrt{X}}}$$

Use of exponents α optimized by Neese and Valeev^63^ for the Ahlrichs basis set family results in $F_{ref}^{DT}=1.038203$ and $F_{ref}^{TQ}=1.137739$ . For CBS extrapolation of the correlation energy, we used the functional expression proposed by Truhlar^64^

7 $$E^{X}=E^{\mathrm{CBS}}+B\cdot X^{\beta }$$

which leads to a two-point CBS extrapolation energy

8 $$E_{\mathrm{corr}}^{\mathrm{CBS}}=\frac{X^{\beta }\cdot E_{\mathrm{corr}}^{X}-Y^{\beta }\cdot E_{\mathrm{corr}}^{Y}}{X^{\beta }-Y^{\beta }}$$

This is equivalent to eq 3 with

9 $$F_{\mathrm{corr}}=\frac{Y^{\beta }}{Y^{\beta }-X^{\beta }}$$

and, with optimized exponents β,^63^ $F_{\mathrm{corr}}^{\mathrm{DT}}=1.607468$ and $F_{\mathrm{corr}}^{\mathrm{TQ}}=1.740740$ . DLPNO–CCSD­(T_1_)/CBS­(DT)/CPS­(56) extrapolated calculations were feasible for the full molecular systems, while extrapolated CBS­(TQ)/CPS(67) results were obtained for truncated molecular models. Detailed benchmark results against CBS extrapolated CCSD­(T*)-F12 data are provided as Supporting Information. In a recent study, Aoalsteinsson and Bjornsson^65^ observed unsatisfactory performance of reference energy CBS extrapolation schemes for DLPNO–CCSD calculations using KS-DFT reference wave functions. They tentatively related this finding to the nonself-consistent nature of the HF reference energies evaluated on KS wave functions and therefore used the reference energy obtained with the larger basis set instead. We note in this context, that the current DLPNO–CCSD implementation in ORCA routinely applies a QRO transformation^58^ for all unrestricted reference orbitals prior to the coupled-cluster calculation. Thus, QRO-transformed reference energies for open-shell cases are generally nonselfconsistent whenever UHF or UKS reference orbitals are used (cf. Table S10). While this might raise some concern as to the generalizability of CBS extrapolation schemes for DLPNO–CCSD reference energies, we find no significant differences in pertinent test calculations (Table S11).

### CCSD­(T*)-F12b Calculations

For benchmarking and ONIOM calculations (cf. below) on truncated smaller molecular models, explicitly correlated coupled-cluster CCSD­(T*)-F12b^66^ single-point energy calculations with the cc-pV­{D,T}­Z-F12 ^67^ (aug-cc-pV­{D,T}­Z-PP for Re)^68^ basis sets were performed with MOLPRO 2024.^69–71^ Perturbative triples contributions improved toward the complete basis set limit via F12-scaling^72^ and denoted as (T*), were obtained by employing the scale factor *E*_corr_(MP2-F12)/*E*_corr_(MP2). The corresponding triple-ζ auxiliary fit basis sets were used, i.e., the JKfit set^73^ for the Fock and exchange integrals, the MP2fit^74^ set for density fitting, and the OptRI set^75,76^ for nonmetal atoms along with the triple-ζ JKfit set for Re for the construction of the complementary auxiliary basis set (CABS). For open-shell cases, we used the ROHF/UCCSD­(T*)-F12b implementation in MOLPRO (“rhf; uccsd­(t)-f12”).^77^ Following Hill et al.,^78^ CCSD and (T*) correlation energy contributions were extrapolated separately to the complete basis set limit employing eq 3 with $F_{\mathrm{CCSD}}^{\mathrm{DT}}=1.387834$ and $F_{\left(T^{*}\right)}^{\mathrm{DT}}=1.529817$ .

Because CASSCF/NEVPT2/QDPT calculations indicated pronounced near-degeneracy effects in the electronic structure of **1a** (see above), we performed additional CCSD­(T*) calculations employing PBE0 and BP86 reference wave functions.^79^ However, only minor differences in relative energies result, and in none of the calculations the usual diagnostics ( $J_{1}$ , *t*_1_, and *t*_2_ amplitudes) signaled alarm, indicating that the QDPT near-degeneracy is accidental in nature. In this context, we noted an inconsistency with the default settings in the CCSD­(T*)-F12b implementation in MOLPRO, at least up to version 2024.2: For closed-shell cases, the use of density fitting in the MP2-based evaluation of the CABS contributions is switched off if KS references are used, whereas the unrestricted coupled-cluster code does use density fitting in this case. This led to substantial inconsistencies of up to 5 kcal·mol^–1^ in computed ROKS-UCCSD­(T*)-F12 results, originating in the CABS correction to the reference energy. For consistent results, we thus used the ROKS/UCCSD­(T*)-F12 algorithm of the open-shell coupled-cluster program also for closed-shell calculations. Problems with (T) contributions in closed-shell KS-CCSD­(T) calculations with MOLPRO have been previously reported by Radoń et al., which led the authors to use the open-shell coupled cluster program also for closed-shell cases.^80^

### ONIOM Calculations

Improved relative energies for the full molecular systems were obtained by using a two-layer ONIOM approach. In this extrapolation scheme, a smaller model system containing the electronically demanding regions is separated from the full molecular system (the “real” system), which is too large for treatment at a sufficiently high level (HL) of theory. Based on a description of the real system employing a low-level (LL) method, a mechanical embedding scheme is used to extrapolate the high-level description of the model system to the entire molecule:^81^

10 $$E_{\mathrm{real}}^{\mathrm{HL}}\approx E^{\mathrm{ONIOM}\left(\mathrm{HL}:\mathrm{LL}\right)}=E_{\mathrm{real}}^{\mathrm{LL}}-E_{\mathrm{model}}^{\mathrm{LL}}+E_{\mathrm{model}}^{\mathrm{HL}}$$

The truncated model systems were constructed by replacing the ^*i*^Pr groups of the pincer ligands of **1a** and **2a**, and the ^*t*^Bu groups of Mes*O and Mes*OH by hydrogen atoms placed along the cleaved P–C and C–C bonds. Constrained geometry optimizations were then performed by relaxing only the newly formed P–H and C–H bond lengths, while all pertinent bond angles and torsion angles, as well as the coordinates of all other atoms, were kept fixed. In the present study, we used DLPNO–CCSD­(T_1_)/CBS­(DT)/CPS­(56) as the low-level method for the real system and CCSD­(T*)-F12b/CBS­(DT) as the high-level method for the H-truncated model systems. Above, this level of theory is abbreviated as CC^ONIOM^.

### CASSCF/NEVPT2/QDPT Calculations

Spin–orbit eigenstates for **1a**/**2a** were calculated with the ORCA 6.0.1 program^54–56^ from state-averaged complete active space computations corrected for dynamic correlation by *n*-electron valence state perturbation theory (CASSCF/NEVPT2) calculations,^82–84^ followed by a quasi-degenerate perturbation theory (QDPT) treatment via a full spin–orbit mean field (SOMF) operator.^85^ CASSCF wave functions were optimized employing the ZORA approximation^86–89^ along with the ZORA-def2-TZVP^90^ basis sets (SARC-ZORA-TZVPP for Re). The RIJK algorithm for fitting the Coulomb and exchange integrals was used in conjunction with the def2/JK auxiliary basis sets (*AutoAux* for Re).^91^ The active space comprises the 5d orbitals of the complexes and the most important interactions of the ligand with the metal center. For **1a**, the three occupied p-orbitals of the equatorial chlorine atom and two occupied pincer-ligand/axial chlorine orbitals were considered, which leads to a CAS­(14,10) expansion. For **2a**, the N–Re π-bonding interaction was additionally considered, giving rise to a CAS­(15,11) expansion. The CAS expansions were state-averaged over 5 quintet, 45 triplet, and 50 singlet roots arising from the formal d^4^ configuration of the rhenium­(III) ion (**1a**) and over 10 quartet and 40 doublet roots for the formal d^3^ configuration of the rhenium­(IV) ion (**2a**), respectively.^92^

## Supplementary Material





## Notes

Spectroscopic, magnetic, electrochemical, crystallographic, calorimetric, and computational data (PDF)
XYZ coordinates of computed structures (TXT)
