# Supplementary material for: Evaluating Spin–Orbit Effects on the Thermochemistry of Proton-Coupled Electron Transfer
Source: Inorg Chem. 2025 Dec 22;65(1):791–801. doi: 10.1021/acs.inorgchem.5c05144 (PMC12801299; doi:10.1021/acs.inorgchem.5c05144)
Supplement: Supplementary file 1 [file ic5c05144_si_001.pdf]

Supporting Information for

Evaluating Spin-Orbit Effects on the Thermochemistry of Proton Coupled Electron Transfer

Daniel Delony,[‡,a] Arnd Fitterer,[‡,b] Martin Diefenbach,[c] Florian Wätjen,[a] Sandipan Maji,[a] Serhiy Demeshko,[a] Matthias Otte,[a] Milan Orlita,[d] Vera Krewald,[c] Max C. Holthausen,[*,b] and Sven Schneider[*,a]

[a] Universität Göttingen, Institut für Anorganische Chemie, Tammannstraße 4, 37077 Göttingen, Germany; sven.schneider@chemie.uni-goettingen.de
[b] Goethe-Universität Frankfurt, Institut für Anorganische und Analytische Chemie, Max-von-Laue Str. 7, 60438 Frankfurt am Main, Germany; max.holthausen@chemie.uni-frankfurt.de
[c] TU Darmstadt, Department of Chemistry, Quantum Chemistry, Peter-Grünberg-Straße 4, 64287 Darmstadt, Germany
[d] Laboratoire National des Champs Magnetiques Intenses, 25 Rue des Martyrs, 38042 Grenoble, France

Table of Contents

1. SPECTROSCOPIC DATA **2**
1.1 IR SPECTROSCOPY 2
1.2 UV/VIS/NIR SPECTROSCOPY 3
1.3 NMR SPECTROSCOPY 4
2. SQUID MAGNETOMETRY **7**
3. ELECTROCHEMICAL DATA **8**
4. SINGLE-CRYSTAL X-RAY DIFFRACTION **9**
4.1 [$ReCl_3$(^{H}PNP)][B{C_6H_3(3,5-CF_3)$_2$}$_4$] (**3A**) 9
4.2 [$ReBr_3$(^{H}PNP)][B{C_6H_3(3,5-CF_3)$_2$}$_4$] (**3B**) 10
5. THERMOCHEMICAL EXPERIMENTS **11**
5.1 THERMOCHEMICAL EXPERIMENTS IN CH_2Cl_2 11
5.2 THERMOCHEMICAL EXPERIMENTS IN THF 14
6. COMPUTATIONAL DATA **17**
6.1 PBE0-D3BJ/DEF2-TZVP MOLECULAR STRUCTURES 17
6.2 THERMOCHEMISTRY OF THE H-TRUNCATED MODEL SYSTEM 18
6.3 CONSISTENCY OF THE ONIOM-APPROACH 19
6.4 THERMOCHEMISTRY OF THE FULL MOLECULAR SYSTEM 19
6.6 TOTAL ENERGIES OF THE H-TRUNCATED MODEL SYSTEM 24
6.7 TOTAL ENERGIES OF THE FULL MOLECULAR SYSTEM 26
7. REFERENCES **27**

1. Spectroscopic Data

1.1 IR Spectroscopy

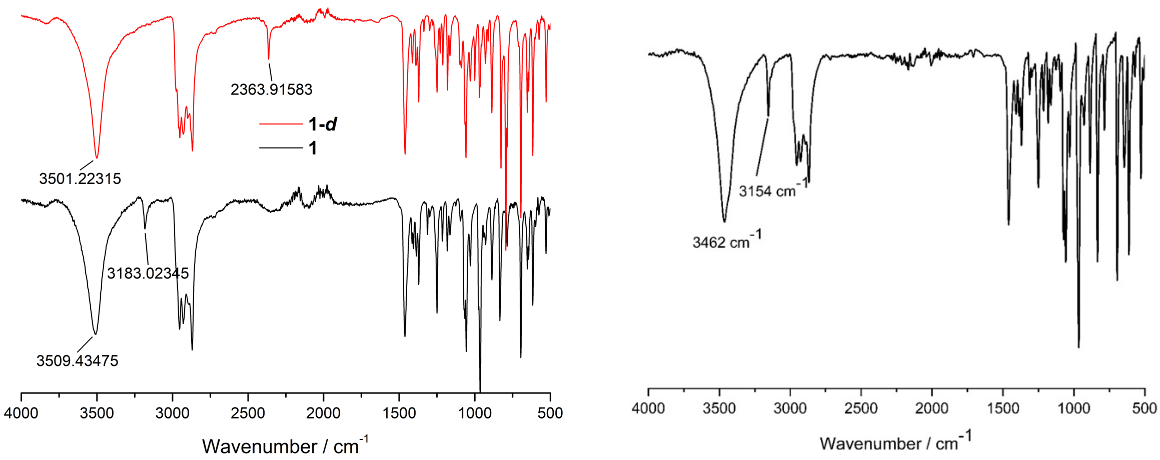

Figure S1. *Left*: Comparison of the ATR-IR spectra of **1a** (black trace) and of **1a** after H/D exchange of the N–H proton (red trace). *Right*: ATR-IR spectrum of **1b**.

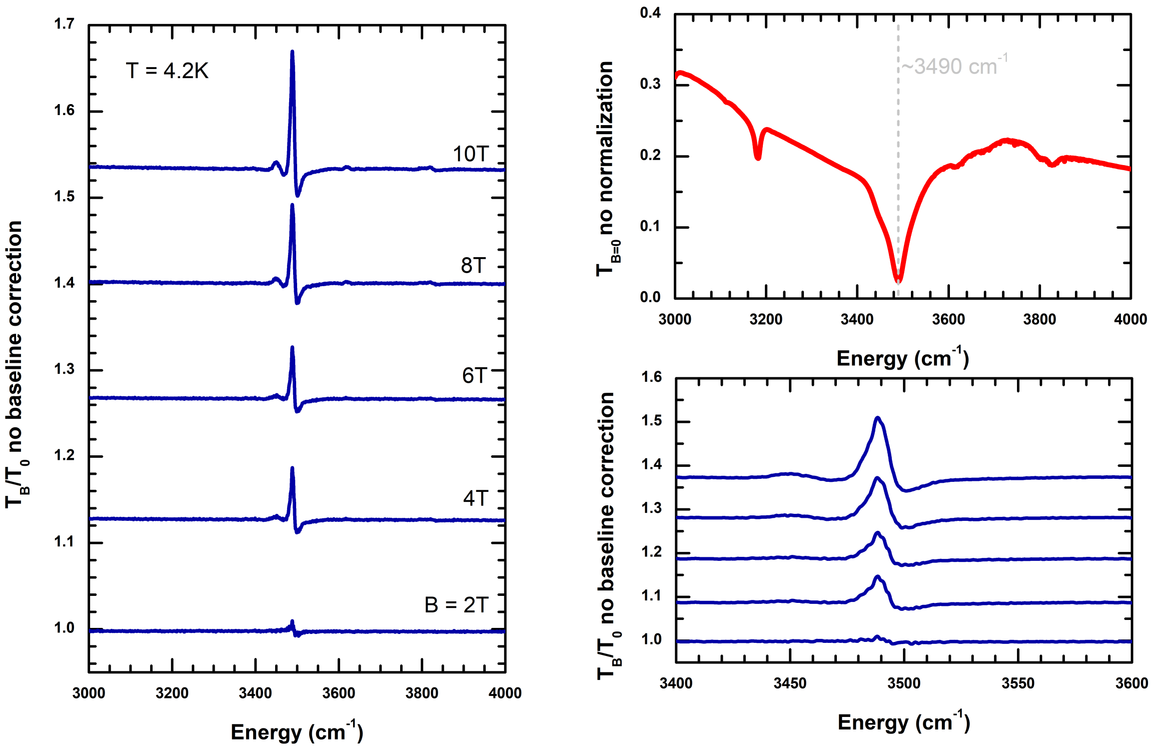

Figure S2. Field dependent magnetic IR data of **1a**. *Left*: Transmission spectra with applied field (T_B) divided by zero field transmission spectrum (T_0). *Top right*: Zero field transmission IR spectrum at 4.2 K. *Bottom right*: Zoom in on the electronic transition band.

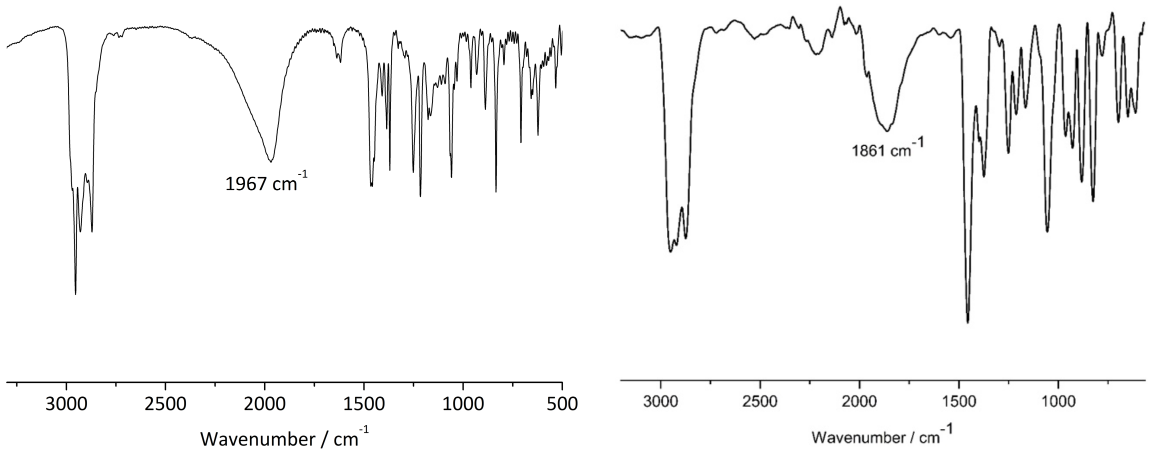

Figure S3. *Left*: IR spectrum of **2a** in KBr. *Left*: IR spectrum of **2b** in KBr.

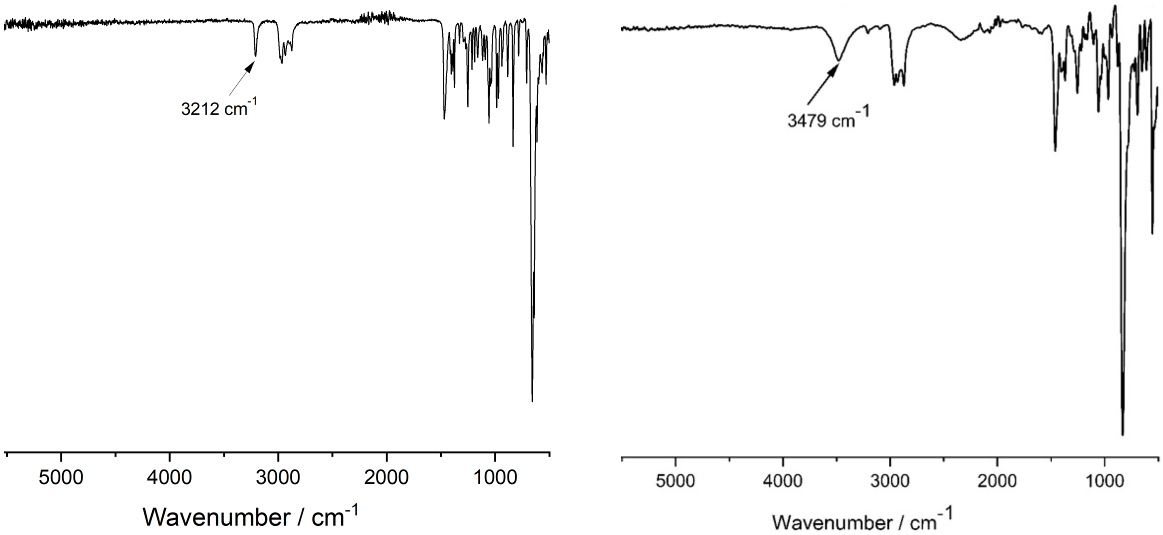

Figure S4. *Left*: ATR-IR spectrum of **3a**. *Right*: ATR-IR spectrum of **3b**.

1.2 UV/Vis/NIR Spectroscopy

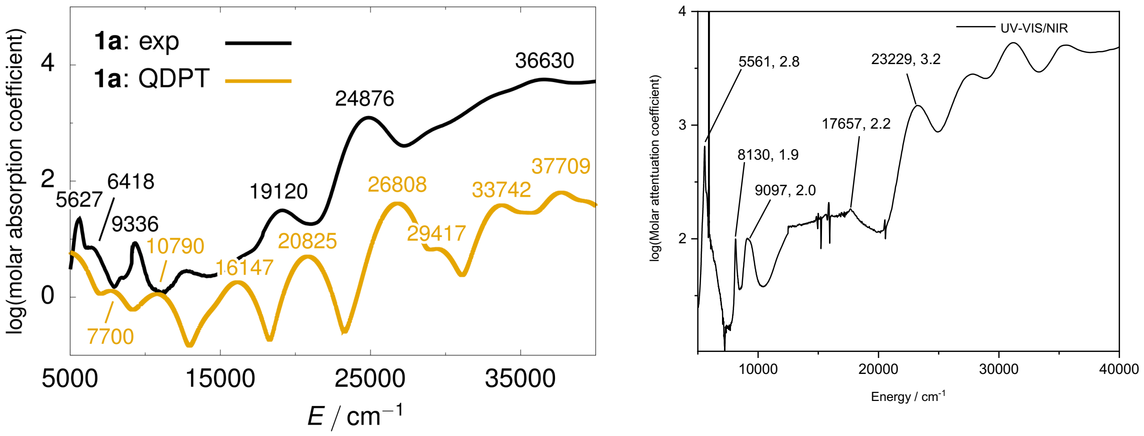

Figure S5. UV/Vis/NIR spectra of amine complexes **1a** (*left*) and **1b** (*right*) in THF solution (black lines). The computed spectrum for **1a** (magenta line) was obtained at the CASSCF/NEVPT2-QDPT level of theory (see computational section).

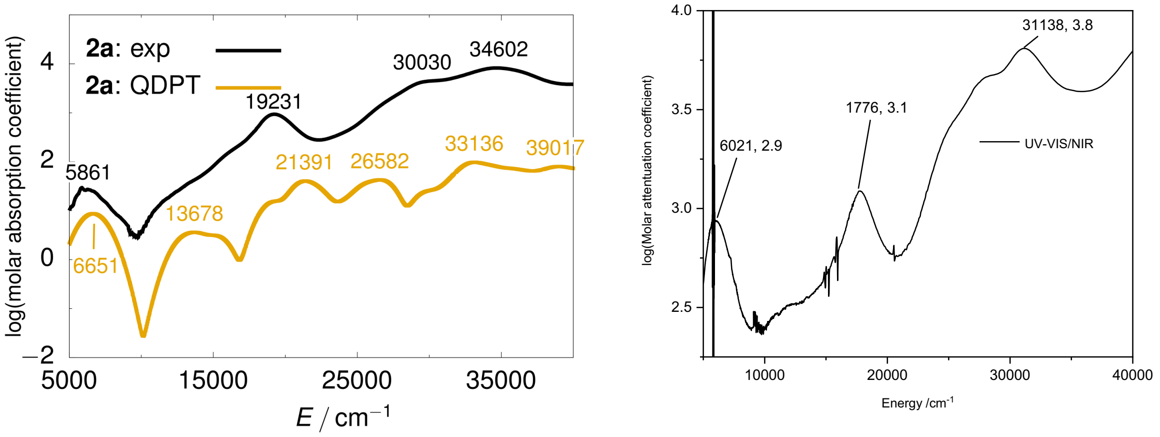

Figure S6. UV/Vis/NIR spectra of amido complexes **2a** (*left*) and **2b** (*right*) in THF solution (black lines). The computed spectrum for **2a** (magenta line) was obtained at the CASSCF/NEVPT2-QDPT level of theory (see computational section).

Table S1. Comparison of the spectroscopic and magnetic data of **1a** and **2a** in comparison to CASSCF/NEVPT2/QDPT computed values.

	**1a**		**2a**	
	Experimental	*Computed*]	*Experimental*	*Computed*
$\mu_{eff,RT}$ / μ_B	1.60	1.54	1.86	1.82
UV/Vis / cm^{-1}	24876 (1238)	27907	30030 (4457)	29131
(ε / M^{-1} cm^{-1})	19287 (29)	20914	19231 (935)	18357
NIR / cm^{-1}	9335 (3)	10800		
(ε / M^{-1} cm^{-1})	6421 (7)	7700	6140 (18)	5900
	5622 (21)	6300		
IR / cm^{-1} (ε / M^{-1} cm^{-1})	3513 (n.d.)	3918	1970 (n.d.)	2251

1.3 NMR Spectroscopy

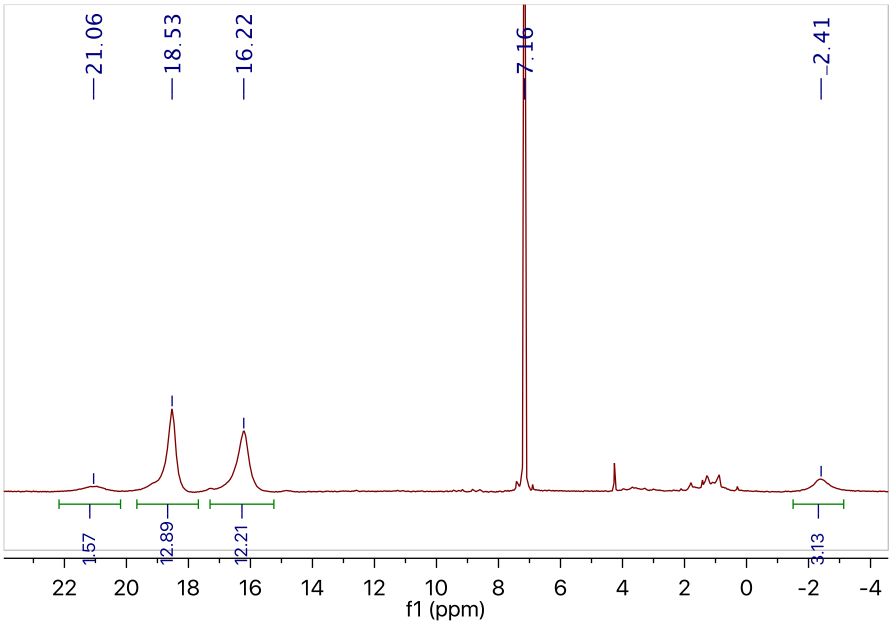

Figure S7. ^1H-NMR spectrum of **2b** in C_6D_6.

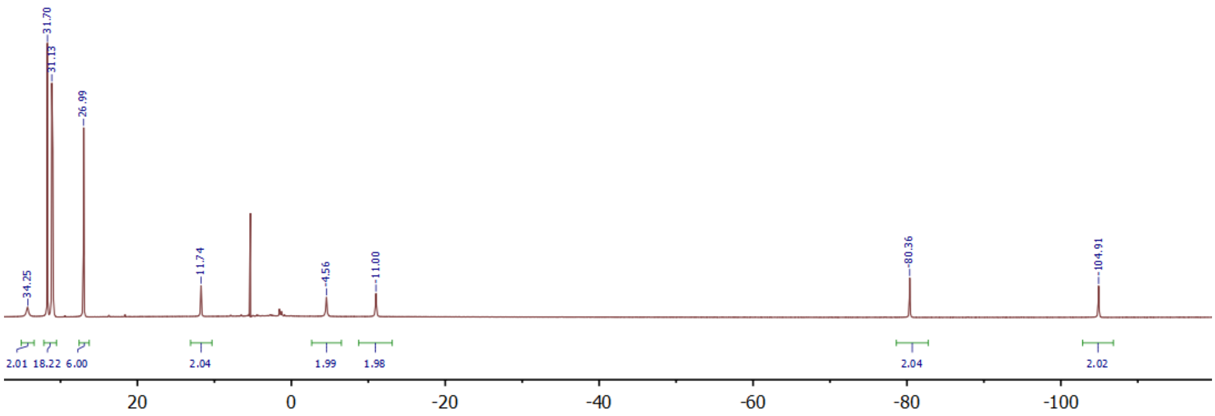

Figure S8. ^{1}H NMR spectrum of **3a** in CD_2Cl_2.

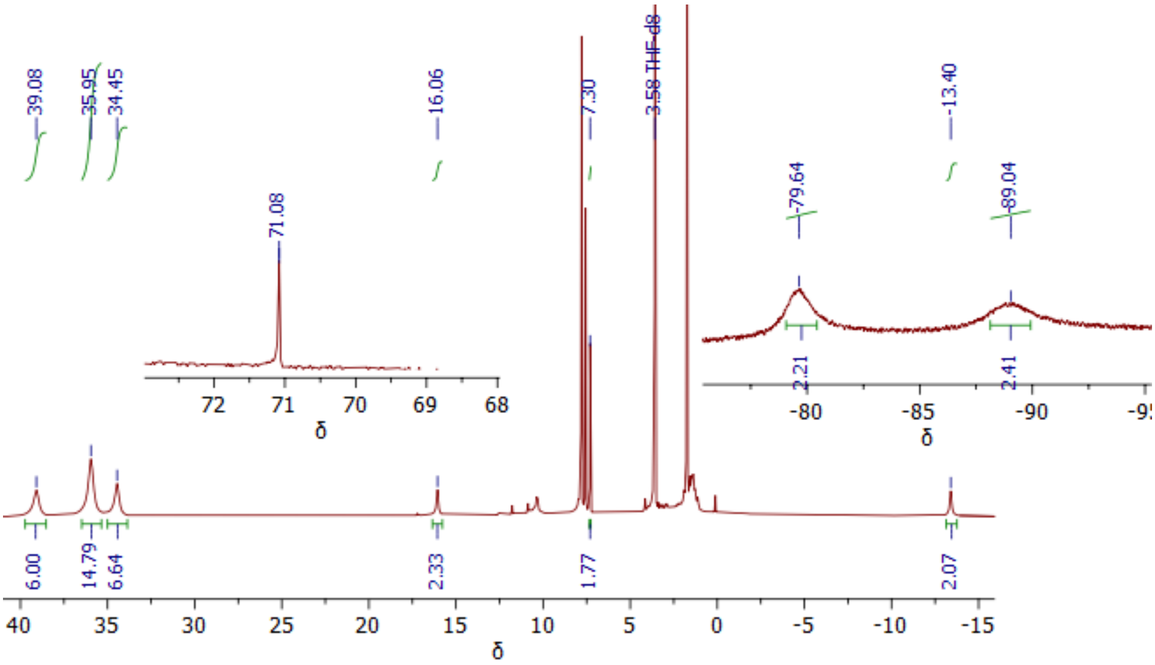

Figure S9. ^{1}H NMR spectrum of **3b** in THF-d_8.

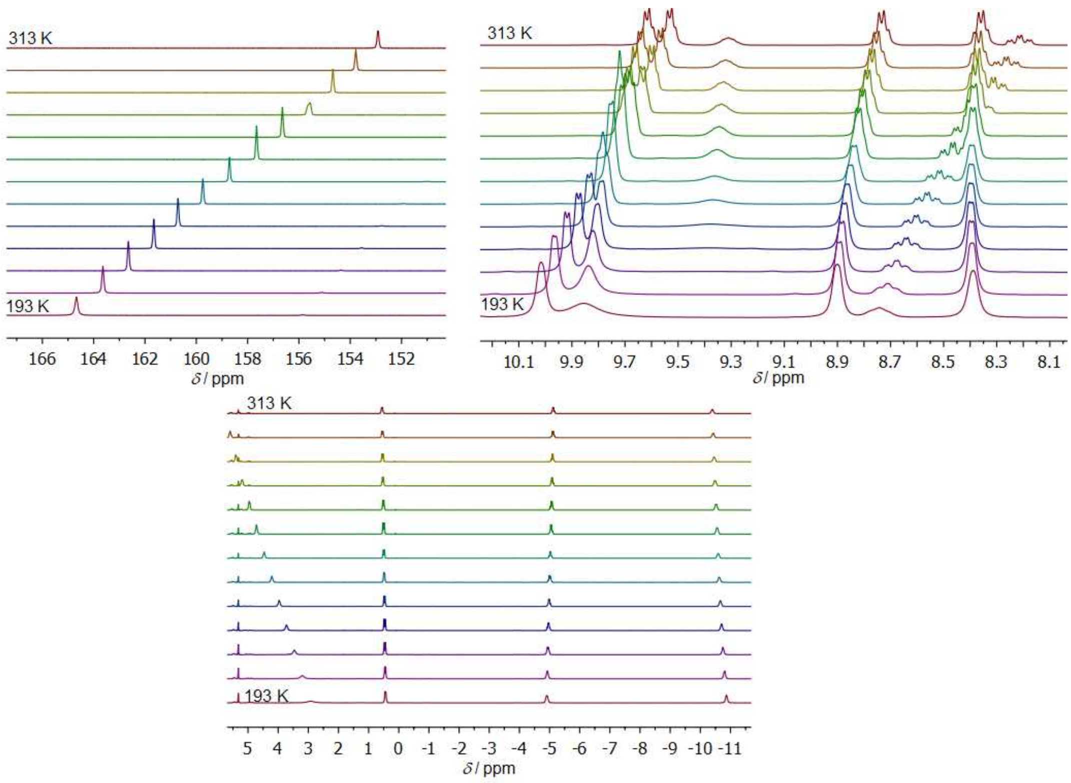

Figure S10. VT ^{1}H NMR spectra of **1a** between –80 °C and +40 °C.

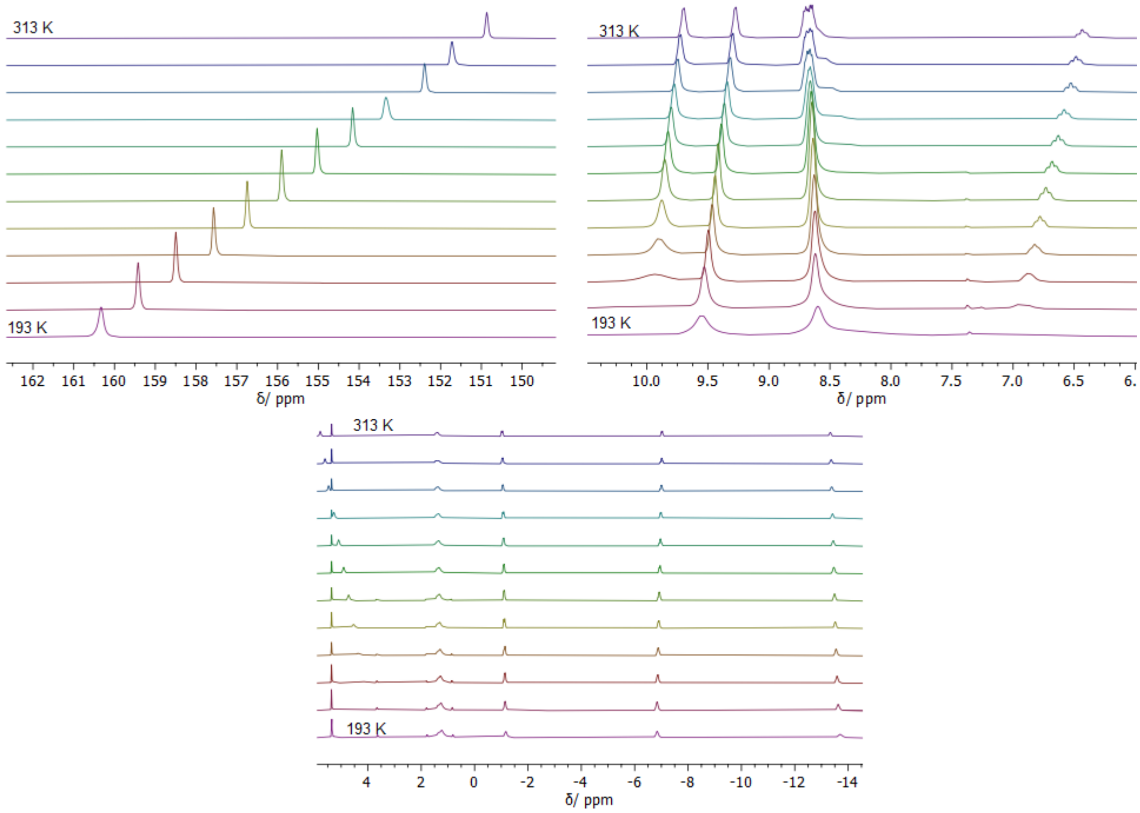

Figure S11. VT ^{1}H NMR spectra of **1b** between –80 °C and +30 °C.

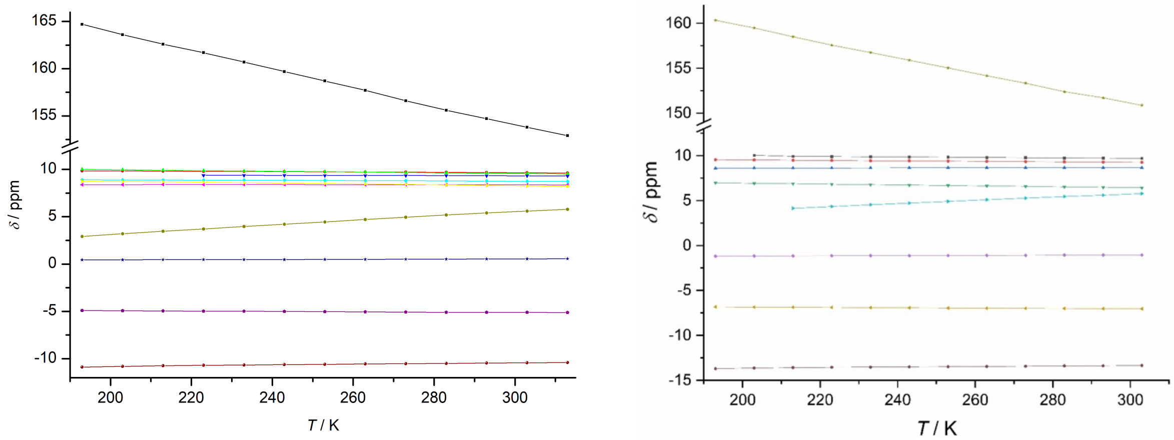

Figure S12. Temperature dependence of the chemical shifts of **1a** (*left*) and **1b** (*right*).

2. SQUID Magnetometry

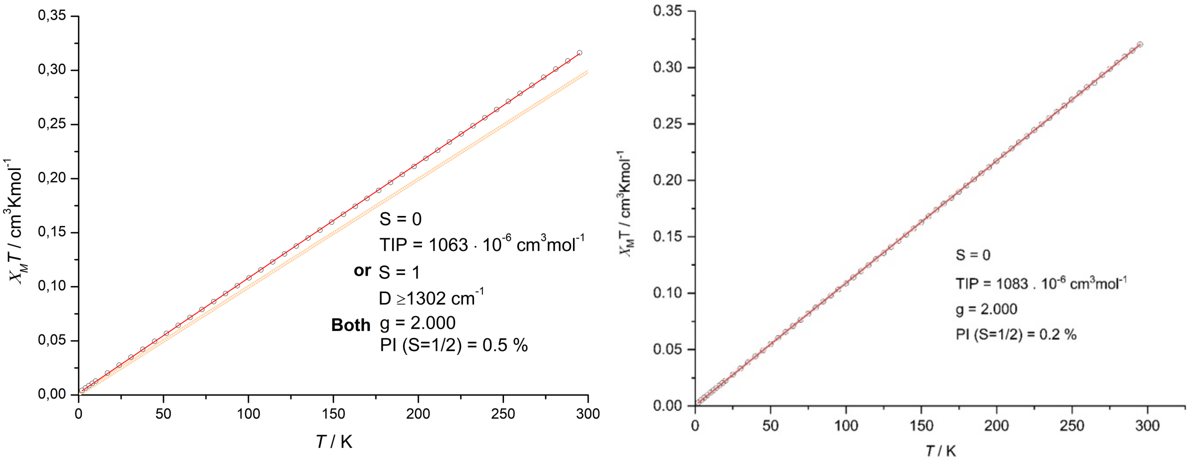

Figure S13. *Left:* Experimental (black circles) and simulated (red line) SQUID magnetometry data of **1a** (0.5 T) and computed magnetic susceptibility from state-averaged CAS(14,10)SCF/NEVPT2/QDPT computations (brown line). *Right*: Experimental (black circles) and simulated (red line) SQUID magnetometry data of **1b** (0.5 T).

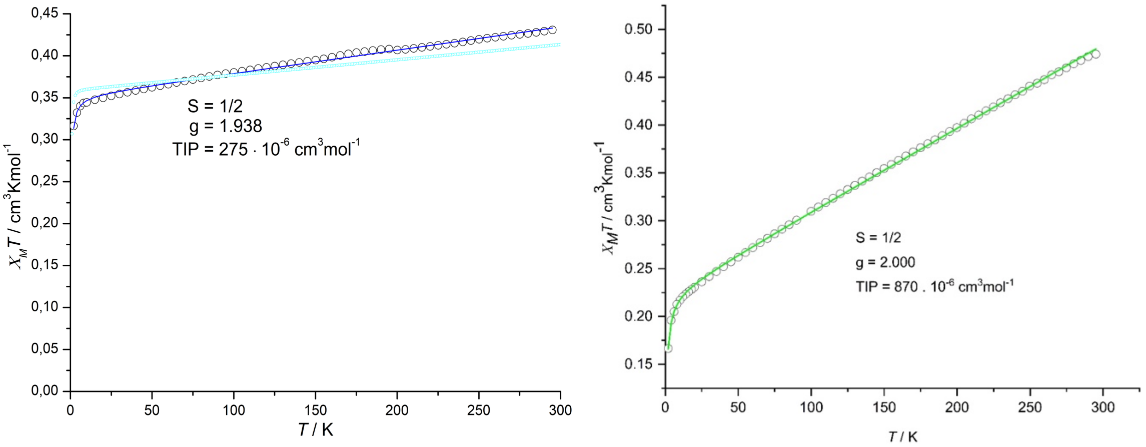

Figure S14. *Left*: Experimental (black circles) and simulated (dark blue line) SQUID magnetometry data of **2a** (0.5 T) and computed magnetic susceptibility from state-averaged CAS(15,11)SCF/NEVPT2/QDPT computations (light blue line). *Right*: Experimental (black circles) and simulated (green line) SQUID magnetometry data of **2b** (0.5 T).

3. Electrochemical Data

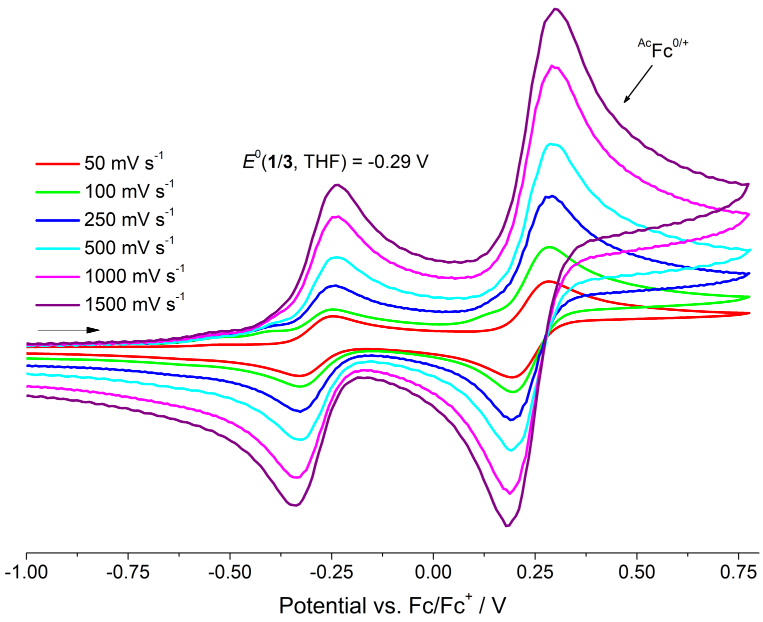

Figure S15. Cyclic Voltammogram of **1a** and acetylferrocene (E^0 = +0.24 V) as internal standard in THF (1 mM; NBu_4PF_6 0.1 M).

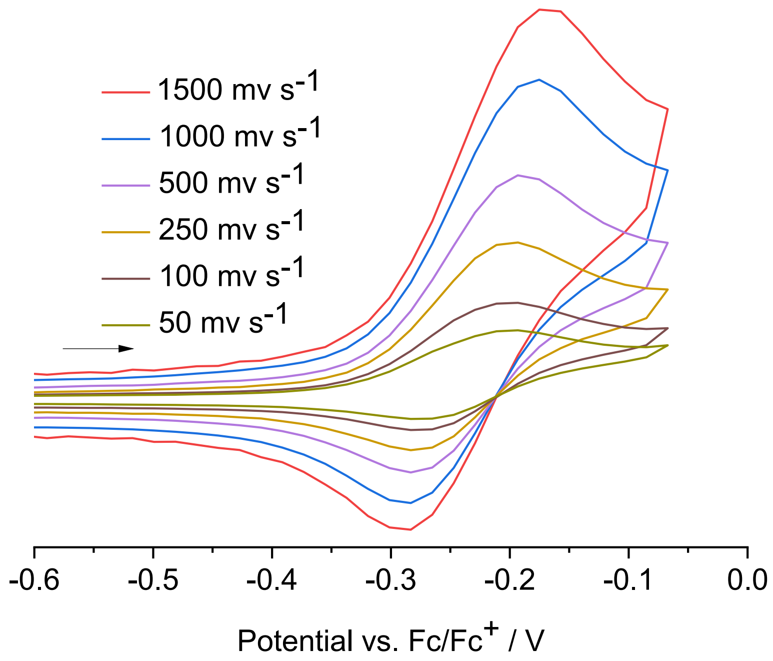

Figure S16. Cyclic Voltammogram of **1b** in THF (1 mM; NBu_4PF_6 0.2 M).

4. Single-crystal X-ray Diffraction

Crystallographic Details

Suitable single crystals for X-ray structure determination were selected from the mother liquor under an inert gas atmosphere and transferred in protective perfluoro polyether oil on a microscope slide. The selected and mounted crystals were transferred to the cold gas stream on the diffractometer. The diffraction data were obtained at 100 K on a Bruker D8 three-circle diffractometer, equipped with a PHOTON III detector and an INCOATEC microfocus source with Quazar mirror optics (Mo-Kα radiation, λ= 0.71073 Å).

The data obtained were integrated with SAINT and a semi-empirical absorption correction from equivalents with SADABS was applied. The structure was solved and refined using the Bruker SHELX 2014 software package.[1] All non-hydrogen atoms were refined with anisotropic displacement parameters. All C-H hydrogen atoms were refined isotropically on calculated positions by using a riding model with their U_{iso} values constrained to 1.5 U_{eq} of their pivot atoms for terminal sp^3 carbon atoms and 1.2 times for all other atoms.

4.1 [$ReCl_3$(^{H}PNP)][B{C_6H_3(3,5-CF_3)$_2$}$_4$] (**3a**)

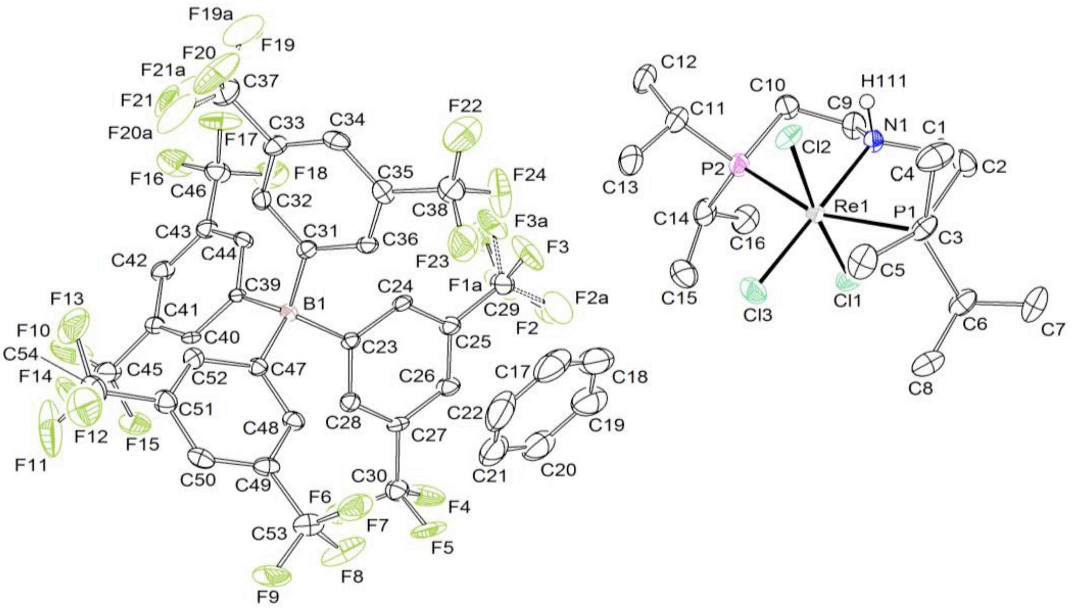

Figure S17. Thermal ellipsoid plot of **3a** with the anisotropic displacement parameters drawn at the 50% probability level. The asymmetric unit contains one complex molecule, one benzene solvent molecule and one BArF$_{24}$ anion with two disordered CF_3 groups. The disordered CF_3 groups were refined in two different positions with population of 0.829(5) on their main domains using PART commands and some restraints and constrains (SADI, RIGU, SIMU, EADP). The N-H hydrogen atom was refined isotropically on a calculated position using AFIX 13 command.

Table S2. Crystal data and structure refinement for **3a**.

Identification code	mo_CW_DD_22719_0m_a (DD-380)	
CCDC Identifier	2498886	
Empirical formula	C54H55BCl3F24NP2Re	
Formula weight	1539.29	
Temperature	100(2) K	
Wavelength	0.71073 Å	
Crystal system	Orthorhombic	
Space group	P21/c	
Unit cell dimensions	a = 14.0923(10) Å	α= 90°
	b = 15.5255(12) Å	β= 103.942(4)°
	c = 29.199(2) Å	γ=90°
Volume	6200.2(8) Å3	
Z	4	
Density (calculated)	1.649 Mg/m3	
Absorption coefficient	2.250 mm^{-1}	
F(000)	3052	
Crystal size	0.297 x 0.206 x 0.118 mm3	
Crystal shape and color	Block, clear intense orange	
Theta range for data collection	2.229 to 26.585 °	
Index ranges	-17<=h<=17, -19<=k<=19, -36<=l<=36	
Reflections collected	65141	

Independent reflections	12767 [R(int) = 0.1000]
Completeness to theta = 25.242°	99.4%
Refinement method	Full-matrix least-squares on F2
Data / restraints / parameters	12767 / 120 / 834
Goodness-of-fit on F2	1.023
Final R indices [I>2sigma(I)]	R1 = 0.0422, wR2 = 0.0946
R indices (all data)	R1 = 0.0693, wR2 = 0.1059
Largest diff. peak and hole	1.206 and −1.072 eÅ^{-3}

4.2 $[ReBr_3(^{H}PNP)][B\{C_6H_3(3,5\text{-}CF_3)_2\}_4]$ (**3b**)

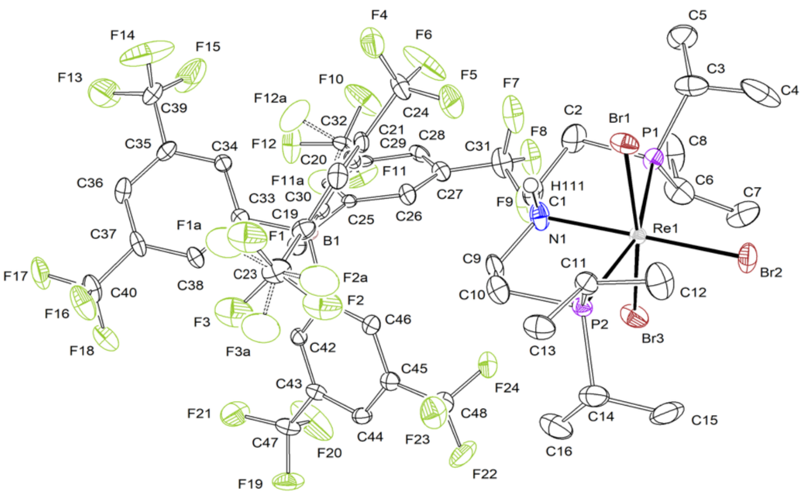

Figure S18. Thermal ellipsoid plot of **3b** with the anisotropic displacement parameters drawn at the 50% probability level. The asymmetric unit contains one complex molecule and one counter ion possessing two disordered CF_3 groups. The disordered counter ion was refined with population of 0.69(9) and 0.64(3) on the main domain using some constraints (EADP). The N-H hydrogen atoms was calculated and constraint to 0.9 Å by using DFIX and isotropically refined.

Table S3. Crystal data and structure refinement for **3b**.

Identification code	SM_220430_MO (SM-36)	
CCDC Identifier	2498887	
Empirical formula	C48H49BBr3F24NP2Re	
Formula weight	1594.56	
Temperature	100(2) K	
Wavelength	0.71073 Å	
Crystal system	Triclinic	
Space group	P-1	
Unit cell dimensions	a =10.2260(5) Å	α= 91.584(3)°
	b = 15.9397(8) Å	β= 103.683(3)°
	c = 18.0318(8) Å	γ=99.175(3)°
Volume	2812.7(2) Å3	
Z	2	
Density (calculated)	1.883 Mg/m3	
Absorption coefficient	4.463 mm^{-1}	
F(000)	1550	
Crystal size	0.174 x 0.044 x 0.014 mm3	
Crystal shape and color	Needle, clear intense purple	
Theta range for data collection	2.080 to 26.429 °	
Index ranges	-12<=h<=12, -19<=k<=19, -22<=l<=22	
Reflections collected	130433	
Independent reflections	11543 [R(int) = 0.0718]	
Completeness to theta = 25.242°	100%	
Refinement method	Full-matrix least-squares on F2	
Data / restraints / parameters	11543 / 1 / 738	
Goodness-of-fit on F2	1.242	
Final R indices [I>2sigma(I)]	R1 = 0.0544, wR2 = 0.1016	
R indices (all data)	R1 = 0.0648, wR2 = 0.1075	
Largest diff. peak and hole	1.841 and −1.582 eÅ^{-3}	

5. Thermochemical Experiments

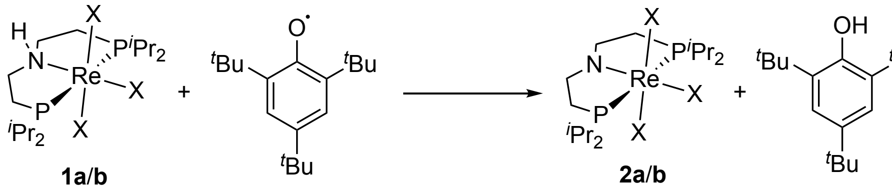

The PCET thermochemistry of **1a** (X = Cl) and **1b** (X = Br) with Mes*O at 298 K in CH_2Cl_2 and THF was derived with two different approaches. For **1a**, he reaction enthalpy (ΔH_r) in CH_2Cl_2 could be directly determined by isothermal titration calorimetry (ITC) upon titration with Mes*O, as described in section 7.1. This approach was not successful for **1b** in CH_2Cl_2 as well as for both complexes in THF due to overoxidation by the titrant. The PCET thermochemistry of **1a** and **1b** in THF was therefore derived via a thermochemical Hess cycle ('square scheme'), providing (ΔG_r) as described in section 7.2.

5.1 Thermochemical experiments in CH_2Cl_2

2,4,6-Tris-*tert*-butyl-phenoxyradical (Mes*-O) was used as titrant with the experimental conditions shown in Table S6. Figures S19-S24 show the thermograms and integrated titration curves. Greyed out points were not considered in the fitting process. The first data point was generally neglected due to the dilution effect within the pre-experiment equilibration time. The heat of reaction (dH) and the stoichiometry (n) were determined by fitting to an independent model. Due to the strongly exothermic nature of reaction, the equilibrium constant (K_a) and free reaction energy (ΔG_r) could not be determined. The results of the three independent runs are summarized in Table S5.

Table S4. ITC measurement details.

	1st/2nd Run	3rd Run
Concentration and volume of **1a** [mM / µL]	0.836/950	1.69/950
Concentration and volume of Mes*-O [mM / µL]	6.54/250	12.9/250
Injection Steps/Injected Volume [µL]	14/16@10	16@10
Waiting time between two additions [s]	200[a]	200[b]
Stirring rate [rpm]	350	350
Equilibration time before start of experiment[s]	1200	1200

[a] The injection time was raised from injection 8 on by 50 s up to 400 s at injection 11 and 250 s from injection 12 due to slower reaction kinetics around the equilibrium point.
[b] Same as [a], except injection 12 (400s) and injection 13 (250 s).

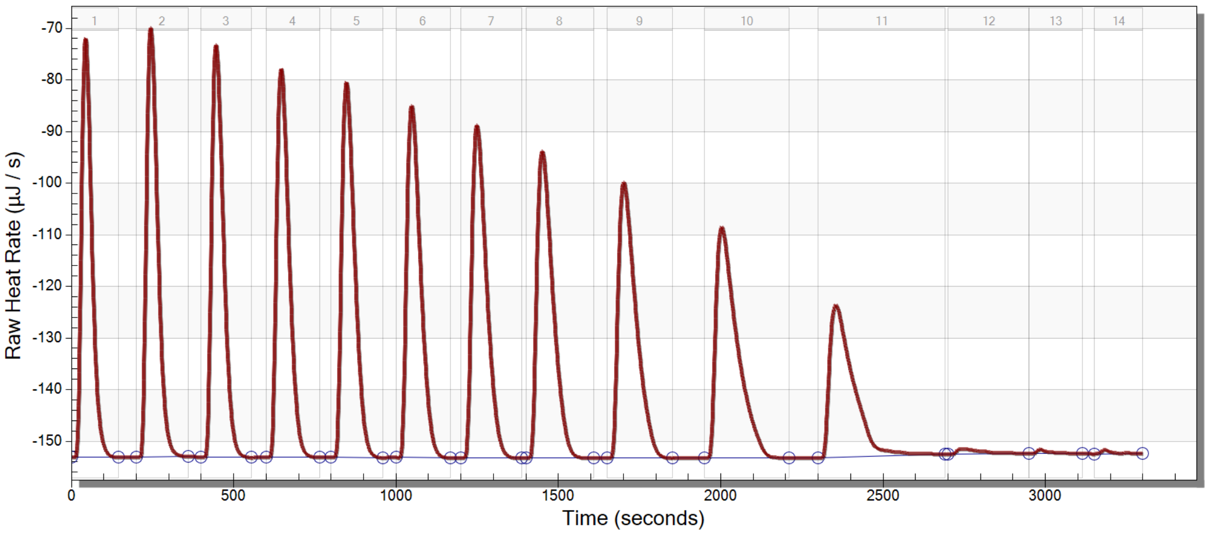

Figure S19. Thermogram of the 1st titration of **1** with Mes*O at 298 K in CH_2Cl_2.

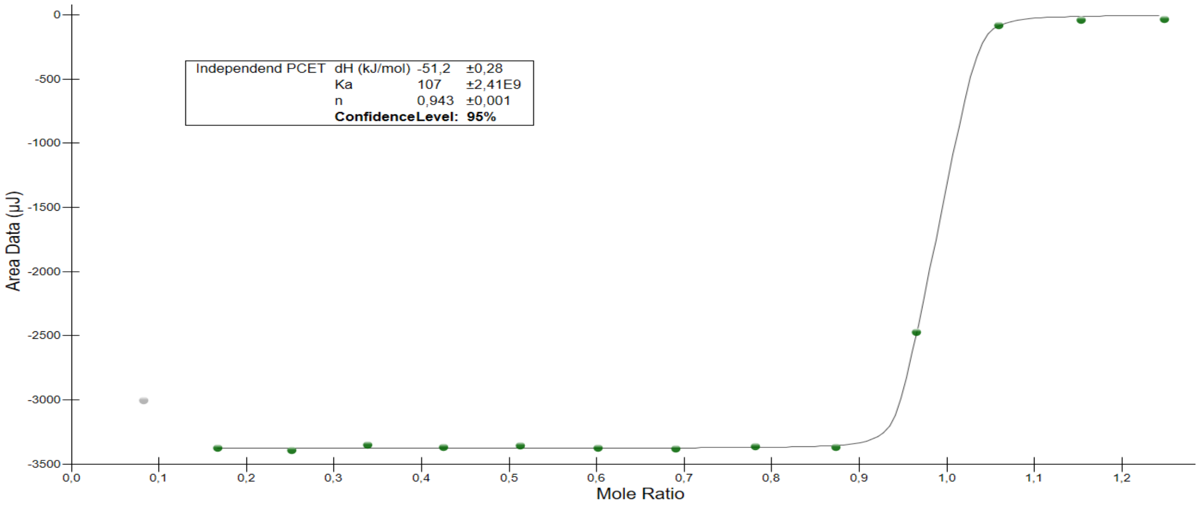

Figure S20. Integrated thermogram of the 1st titration of **1** with Mes*O at 298 K in CH_2Cl_2.

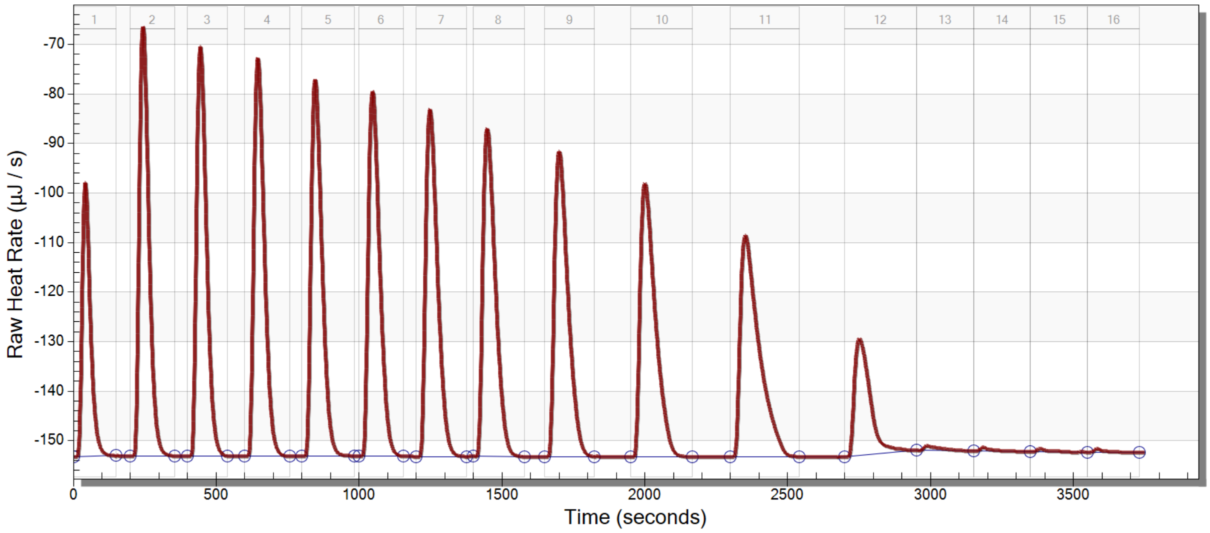

Figure S21. Thermogram of the 2nd titration of **1** with Mes*O at 298 K in CH_2Cl_2.

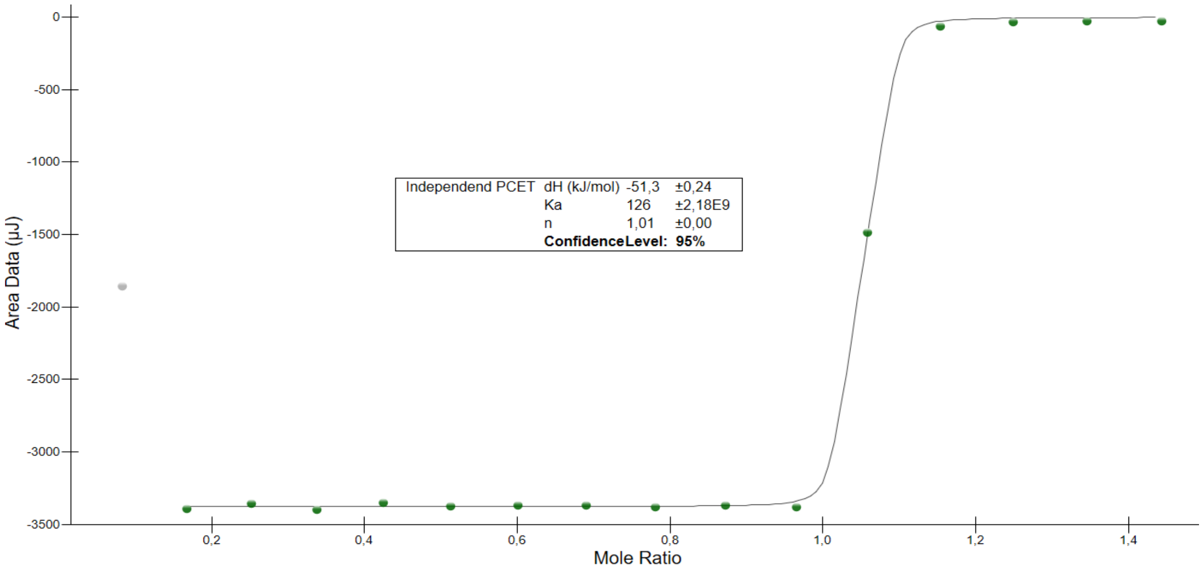

Figure S22. Integrated thermogram of the 2nd titration of **1** with Mes*O at 298 K in CH_2Cl_2.

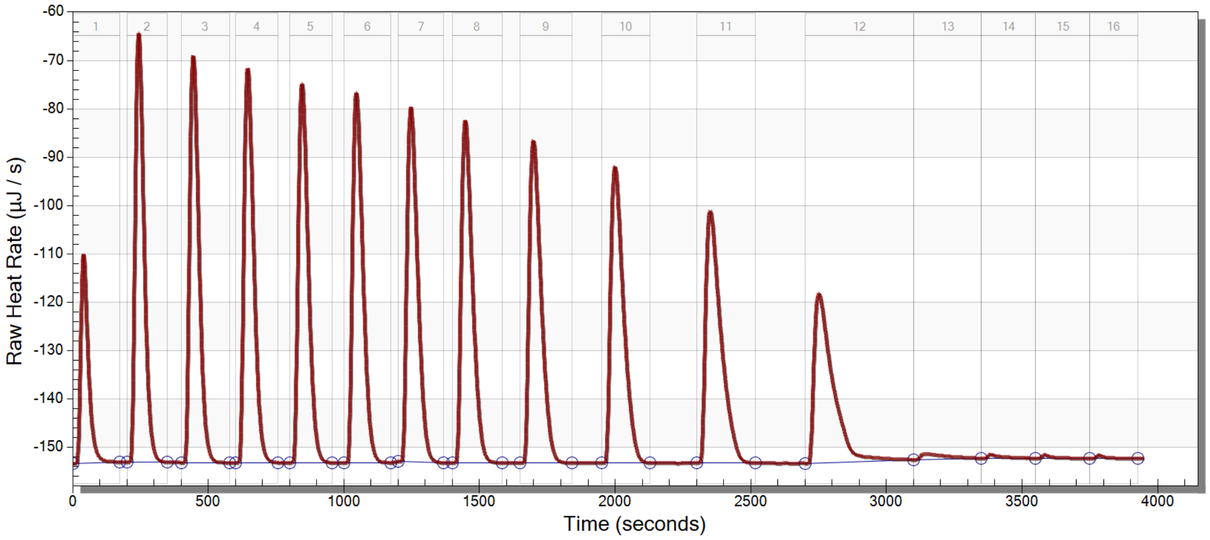

Figure S23. Thermogram of the 3rd titration of **1** with Mes*O at 298 K in CH_2Cl_2.

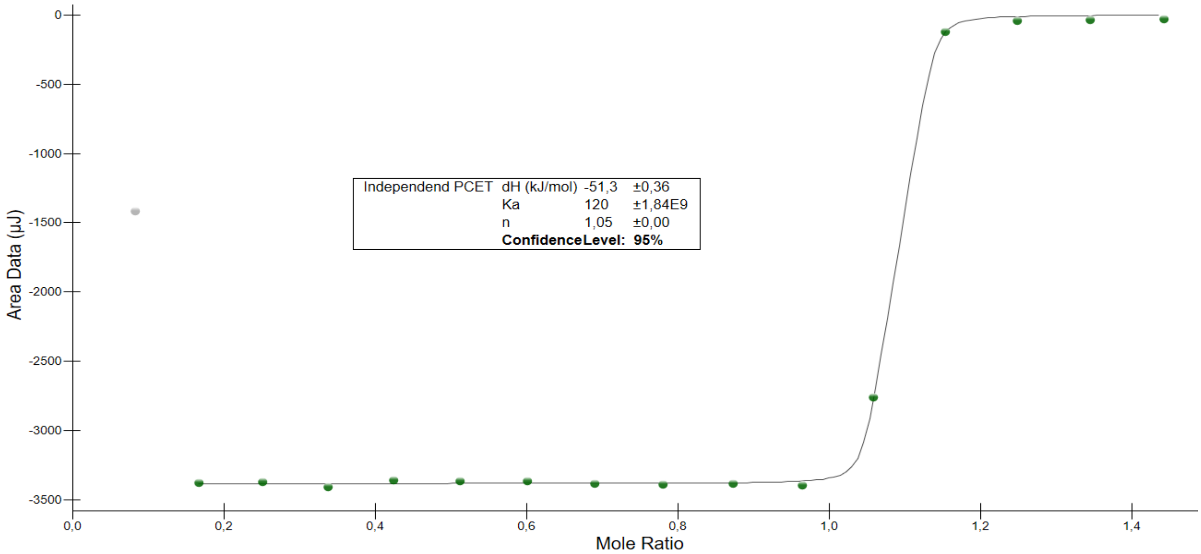

Figure S24. Integrated thermogram of the 3rd titration of **1** with Mes*O at 298 K in CH_2Cl_2.

Table S5. Summary of the ITC results.

ΔH_r [kJ·mol^{-1}]	Error at 95 % confidence	n value	Corrected Error
**–51.2**	±0.28	0.943	±0.30
**–51.3**	±0.24	1.01	±0.24
**–51.3**	±0.36	1.05	±0.38

$$\Delta H^{298\,\mathrm{K}} = -51.2 \pm 0.3\ \mathrm{kJ\ mol^{-1}} = \ 12.2\ \pm 0.1\ \mathrm{kcal\ mol^{-1}}$$

5.2 Thermochemical experiments in THF

The reaction free energies (ΔG_r) of the PCET reactions of **1a**/**b** with Mes*O in THF were derived indirectly via a thermochemical cycle. For this purpose, the oxidation potentials of **1a**/**b** were derived by CV (see section 5) and the pK_a of the oxidation products, [ReCl$_3$(^{H}PNP)]SbF$_6$ (**3a**) and ReBr$_3$(^{H}PNP)]BAr$^F{}_4$ (**3b**), determined by calorimetric titration with pyridine (for **3a**) and lutidine (for **3b**) as bases, respectively. The experimental conditions are shown in Table S6. Figures S25-S28 show the thermograms and integrated titration curves at 298 K. Greyed out data points were not considered in the fitting process. The first data point was generally neglected due to the dilution effect within the pre-experiment equilibration time. The thermochemical parameters (heat of reaction (dH), reaction free energy (dG), equilibrium constant (K_a), and stoichiometry (n)) were determined by fitting to an independent model.

Table S6. ITC measurement details.

	**3a**	**3b**
Concentration and volume of titrand [mM / µL]	1.22/950	1.40/950
Concentration and volume of base [mM / µL]	12.4/250	12.3/250
Injection Steps/Injected Volume [µL]	48@5	50@5
Waiting time between two additions [s]	350[a]	350
Stirring rate [rpm]	350	350
Equilibration time before start of experiment[s]	2700	2040

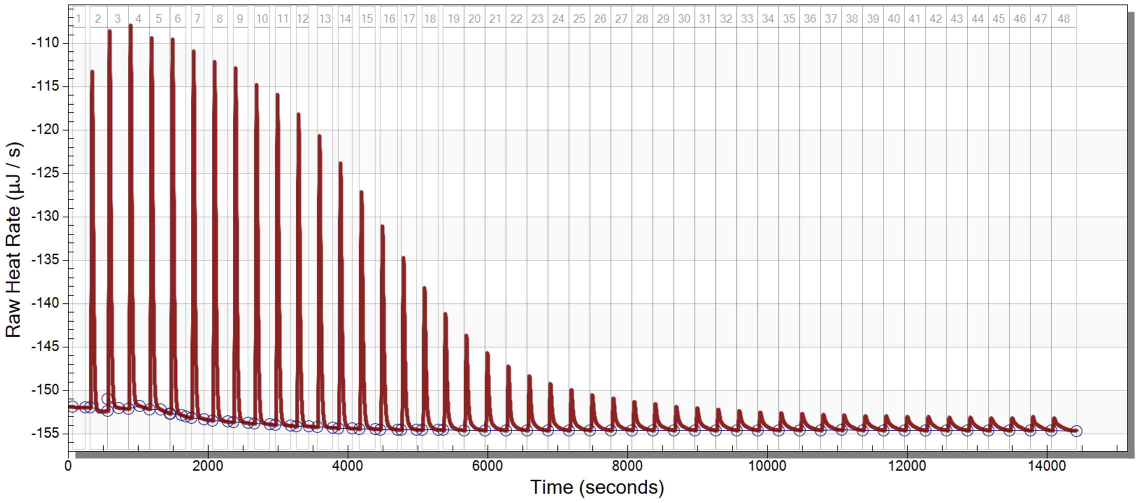

Figure S25. Thermogram of the titration of **3a** with pyridine at 298 K in THF.

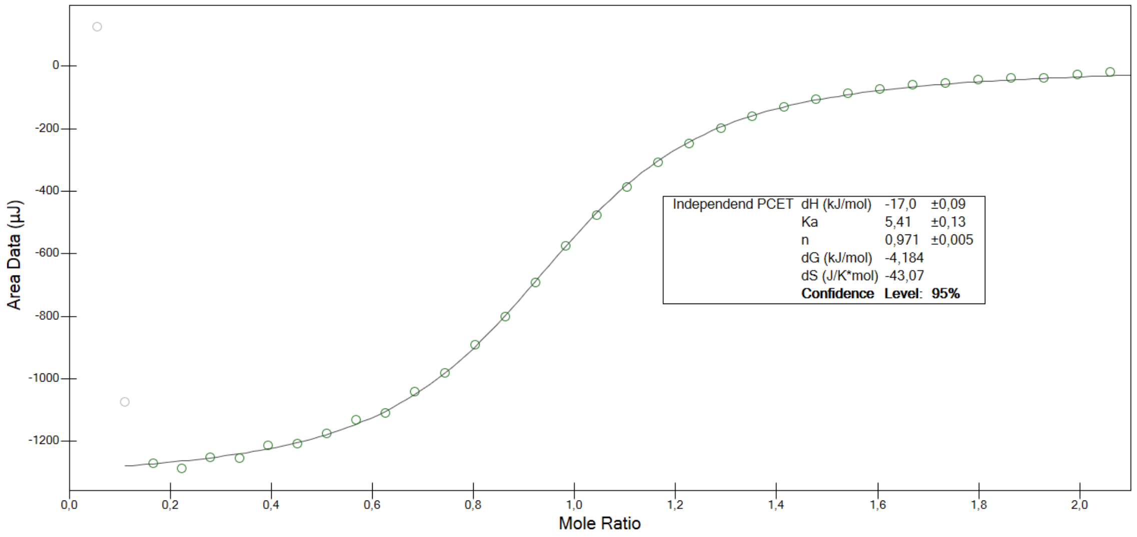

Figure S26. Integrated thermogram of the titration of **3a** with pyridine at 298 K in THF.

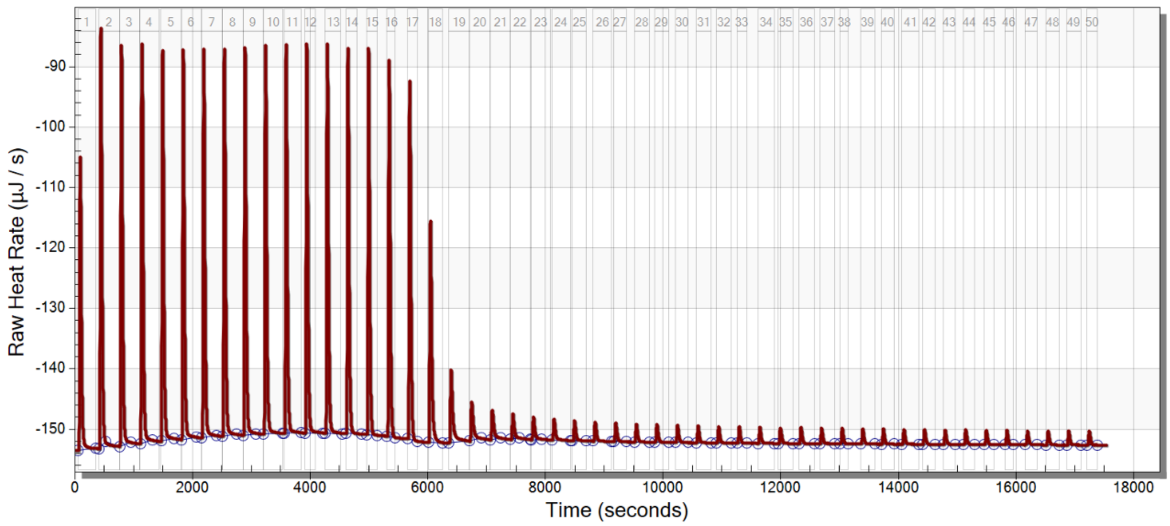

Figure S27:. Thermogram of the titration of **3b** with lutidine at 298 K in THF.

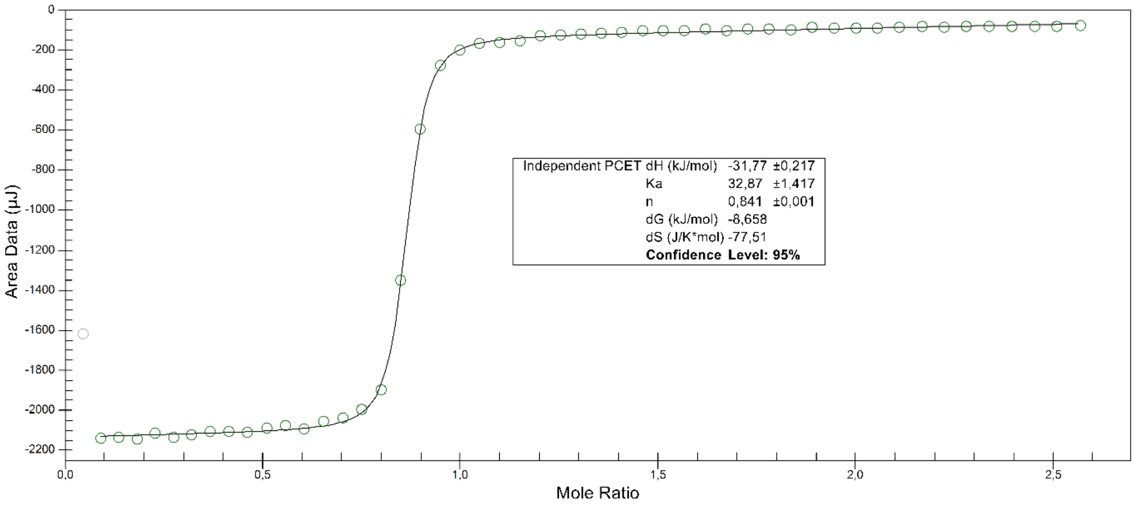

Figure S28. Integrated thermogram of the titration of **3b** with Lutidine at 298 K in THF.

The pK_a values of **3a** and **3b** in THF were obtained from the titration results, using reported pK_a values of pyridine (pK_a = 5.5) and lutidine (pK_a = 7.2) in THF.[2] Furthermore, the Fuoss equation was applied to correct for ion-pairing effects.[3] The radii of **3a**$^+$ (r = 4.96 Å), and SbF_6^- (r = 5.07 Å) were obtained from the molecular structure using single crystal X-ray diffraction. The radius of the pyridinium ion (r = 2.80 Å) was determined from a DFT computed structure. Ion-pairing corrections summed up to ΔpK_D = 0.59, which were also applied to the bromo complex **3b** for simplicity. The following corrected pK_a values were obtained:

pK_a(**3a**) = 5.5 − log 5.4 + 0.59 = 5.4 ± 0.1
pK_a(**3b**) = 7.2 – log 32.9 + 0.59 = 6.3 ± 0.1

The N–H bond dissociation free energies (*BDFE*) of **1a** and **1b** were derived from a thermochemical square scheme (Scheme S29), using the standard oxidation potentials of **1a** (E^0(**3a**/**1a**) = –0.29 ± 0.01 V) and **1b** (E^0(**3b**/**1b**) = –0.22 ± 0.01 V) in THF (see section 5), as well as the respective calorimetrically derived pK_a values in THF (pK_A(**3a**) = 5.4 ± 0.1; pK_A(**3b**) = 6.3 ± 0.1). Calculation of *BDEF*s was carried out using Bordwell's equation (eq. 1), using the free energy of the hydrogen atom in THF reported by Mayer and co-workers (C_G(THF) = 59.9 ± 2 kcal·mol^{-1}).[4] The following N-H *BDFE*s were obtained:

BDFE(**1a**) = 60.5 ± 2.4 kcal mol^{-1}.
BDFE(**1b**) = 63.5 ± 2.4 kcal mol^{-1}.

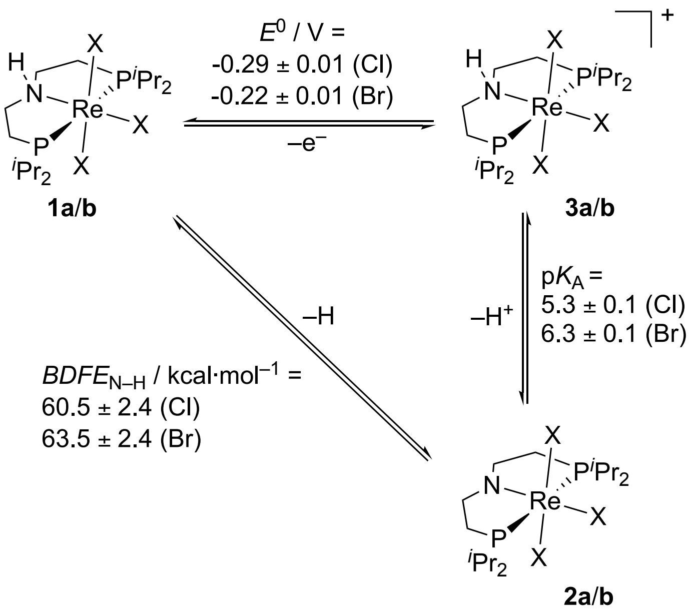

Figure S29. Thermochemical square scheme for the determination of the N-H *BDFE*s of **1a** (X = Cl) and **1b** (X = Br).

Using the reported O-H *BDFE* of Mes*OH in THF ($BDFE_{O–H}$ = 74.4 ± 1 kJ mol^{-1}),[4] the following reaction free enthalpies for the PCET reactions of **1a**/**b** with Mes*O were calculated:

$$\Delta G^{298\,\mathrm{K}}(1a) = -13.9 \pm 2.6\ \mathrm{kcal\ mol^{-1}}$$

$$\Delta G^{298\,\mathrm{K}}(\mathbf{1b}) = -10.9 \pm 2.6\ \mathrm{kcal\ mol^{-1}}$$

6. Computational Data

6.1 PBE0-D3BJ/def2-TZVP molecular structures

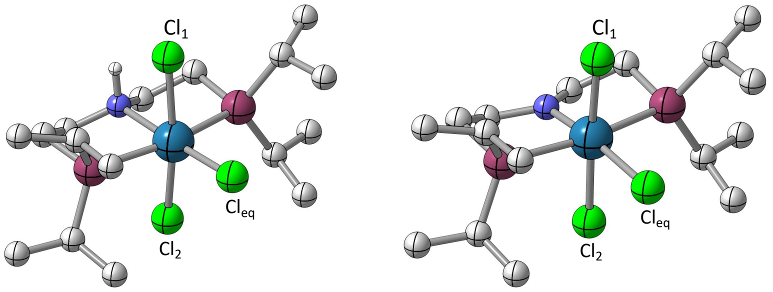

1a **2a**

Figure S30. Representative structures of amine complex **1a** (left) and amide complex **2a** (right) for defining the chlorine atoms Cl_1, Cl_2 and Cl_{eq}. Selected hydrogen atoms omitted for clarity.

Table S7. Structural comparison of the PBE0-D3BJ/def2-TZVP optimized structures with the experimental crystal structures.

Complex	Parameter	PBE0	Experiment[5]
**1a** ^{3}**A“**	Re-N / Å	2.195	2.158 ± 0.018
	Re-Cl_1 / Å	2.390	2.3791 ± 0.0054
	Re-Cl_2 / Å	2.394	2.3725 ± 0.0051
	Re-Cl_{eq} / Å	2.361	2.4117 ± 0.0054
	Re-P[a] / Å	2.421	2.4436 ± 0.0054
	N-Re-Cl_{eq} / °	177.47	177.47 ± 0.54
	Cl_1-Re-Cl_2 / °	169.12	174.73 ± 0.21
	P-Re-P / °	163.32	161.58 ± 0.18
**2a** ^{2}**A“**	Re-N / Å	1.920	1.902 ± 0.021
	Re-Cl_1 / Å	2.322	2.369 ± 0.006
	Re-Cl_2 / Å	2.373	2.379 ± 0.006
	Re-Cl_{eq} / Å	2.432	2.429 ± 0.006
	Re-P[a] / Å	2.429	2.440 ± 0.006
	N-Re-Cl_{eq} / °	172.6	178.7 ± 0.6
	Cl_1-Re-Cl_2 / °	175.54	174.01 ± 0.24
	P-Re-P / °	166.93	163.48 ± 0.24

[a] Mean Re-P bond length.

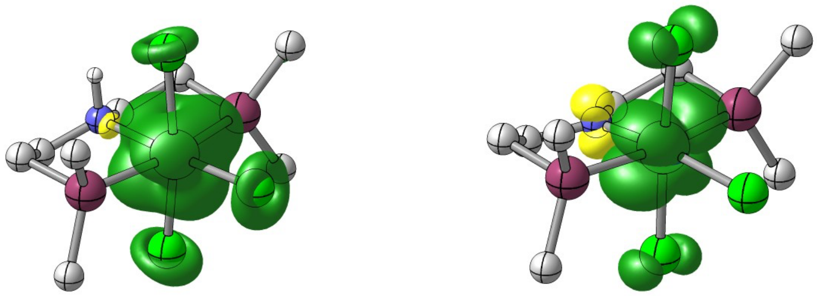

Figure S31. Spin densities of **1a** (^{3}A”, *left*) and **2a** (^{2}A”, *right*), PBE0-D3BJ/def2-TZVP results, isovalues at 0.005 a_0^{-3}. Selected hydrogen atoms and ^{i}Pr-groups not shown for clarity.

Table S8. Mulliken and NBO/NPA spin populations of the different atoms in **1a** and **2a**.

	**1a**		**2a**	
Spin population	Mulliken	NPA	Mulliken	NPA
Re	1.84	1.61	0.96	0.84
∑ P	–0.07	–0.01	–0.06	–0.02
Cl_{eq}	0.08	0.14	0.00	0.00
∑ Cl_{ax}	0.13	0.11	0.16	0.23
N	–0.02	–0.02	–0.10	–0.08

6.2 Thermochemistry of the H-truncated model system

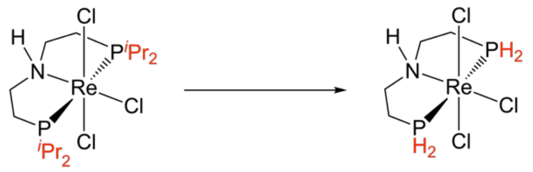

1a **1a^model^**

Table S9 shows a comparison of various coupled cluster approaches, using HF and KS reference wavefunctions and basis set (CBS) extrapolation, as well as CPS extrapolations for DLPNO coupled cluster theory, for spin state energies, the reaction energy and homolytic N–H and O–H dissociation energies for the H-truncated model systems. PBE0-D3BJ/def2-TZVP results show poor agreement in particular for spin state splittings and the O–H dissociation energy, while BP86-D3BJ/def2-TZVP results show fortuitous agreement with the reference data.

Table S9. Spin state energies, reaction energy and N–H/O–H homolytic dissociation energies, computed with explicitly correlated coupled-cluster, DLPNO coupled-cluster and DFT methods. All energies in kcal mol^{-1}.

	$\Delta E_{\mathrm{S-T}}$ (**1a**^{model})	$\Delta E_{\mathrm{D-Q}}$ (**2a**^{model})	ΔE_{r}	$\Delta E_{\mathrm{N-H}}$ (**1a**^{model})	$\Delta E_{\mathrm{O-H}}$ (Phenol)
**HF-CCSD(T*)-F12/CBS(DT)**	**–1.1**	**–11.2**	**–23.4**	**71.3**	**94.7**
PBE0-CCSD(T*)-F12/CBS(DT)	–1.2	–11.3	–24.1	70.5	94.6
BP86-CCSD(T*)-F12/CBS(DT)	–1.0	–10.7	–24.0	70.6	94.6
HF-CCSD(T*)-F12/VDZ	–1.7	–11.8	–24.7	69.5	94.2
PBE0-CCSD(T*)-F12/VDZ	–2.0	–12.1	–24.3	69.8	94.1
BP86-CCSD(T*)-F12/VDZ	–1.9	–12.0	–24.1	69.9	94.1
HF-CCSD(T*)-F12/VTZ	–1.3	–11.4	–23.8	70.8	94.6
PBE0-CCSD(T*)-F12/VTZ	–1.5	–11.5	–24.3	70.2	94.5
BP86-CCSD(T*)-F12/VTZ	–1.4	–11.4	–24.5	70.0	94.5
HF-DLPNO-CCSD(T_1)/CBS(DT)/CPS(56)[a]	0.6	–10.3	–24.1	72.7	96.8
HF-DLPNO-CCSD(T_1)/CBS(TQ)/CPS(56)[a]	0.8	–8.8	–24.8	70.9	95.7
PBE0-DLPNO-CCSD(T_1)/CBS(DT)/CPS(56)[b]	1.0	–9.1	–24.8	–	–
BP86-DLPNO-CCSD(T_1)/CBS(DT)/CPS(56)[b]	1.9	–8.6	–24.6	–	–
HF-DLPNO-CCSD(T_1)/CBS(DT)/CPS(67)[a]	0.3	–10.0	–23.6	72.8	96.4
HF-DLPNO-CCSD(T_1)/CBS(TQ)/CPS(67)[a]	0.4	–9.5	–24.5	71.2	95.7
PBE0-DLPNO-CCSD(T_1)/CBS(TQ)/CPS(67)[b]	0.0	–9.9	–25.3	–	–
BP86-DLPNO-CCSD(T_1)/CBS(TQ)/CPS(67)[b]	0.3	–9.7	–25.0	–	–
PBE0-D3BJ/def2-TZVP	3.5	–6.3	–22.8	68.0	90.0
BP86-D3BJ/def2-TZVP	–0.3	–12.5	–25.2	66.4	91.6

[a] Unrestricted (self-consistent, thus not QRO) HF energies for hydrogen atoms used to compute the O–H and N–H dissociation energies.
[b] O–H and N–H dissociation energies not computed because KS-DLPNO-CCSD(T) energy calculations are not possible for hydrogen atoms with ORCA 6.0.1.

Table S10. SCF-energies (E_{SCF}), reference energies after the QRO transformation (E_{ref}) and DLPNO-CCSD(T_1)/def2-TZVPP/T_{CutPNO}=10^{-5} energies ($E_{\mathrm{DLPNO-CCSD(T1)}}$) for the spin state splitting of **2a**^{model} as well as <S^2> values of the corresponding UHF/UKS wavefunctions.

	<S^2>		$\Delta E_{\mathrm{D-Q}}$ (**2a**^{model})		
Reference orbitals	^{2}**2a**^{model}	^{4}**2a**^{model}	E_{SCF}	E_{ref}	$E_{\mathrm{DLPNO-CCSD(T1)}}$
ROHF	–	–	16.5	16.5	0.0
UHF	1.38	3.80	18.8	30.1	–0.8
ROKS (PBE0)	–	–	–8.2	15.0	–1.6
UKS (PBE0)	0.76	3.77	–6.3	14.8	–1.7
ROKS (BP86)	–	–	–12.7	14.6	–1.2
UKS (BP86)	0.76	3.76	–11.6	14.9	–1.3

Table S11. Comparison of DLPNO-CCSD(T_1)/CBS(DT)/CPS(56) results based on extrapolated reference energies (CBS(DT)) and results obtained using (non-extrapolated) triple-zeta reference energies (def2-TZVPP). All energies in kcal mol^{-1}.

Reference energy	ΔE_{S-T} (**1a**^{model})	ΔE_{D-Q} (**2a**^{model})	ΔE_r	ΔE_{N-H} (**1a**^{model})	ΔE_{N-H} (Phenol)
CBS(DT)	0.6	–10.3	–24.1	72.7	96.8
def2-TZVPP	0.6	–10.3	–24.0	72.8	96.8

6.3 Consistency of the ONIOM-approach

Table S12 shows a validation of the ONIOM-approach using HF/def2-TZVPP as low level method and HF-DLPNO-CCSD(T_1)/CBS(DT)/CPS(56) as high level method: High level results obtained for the real system are met within 0.5 kcal mol^{-1} for the spin state splittings.

Table S12. Comparison of HF-DLPNO-CCSD(T_1), HF and ONIOM(HF-DLPNO-CCSD(T_1):HF) results for the full system and the H-truncated model system. All energies in kcal mol^{-1}.

		ΔE_{S-T} (**1a**)	ΔE_{D-Q} (**2a**)
HF/def2-TZVPP	low level (model system)	22.7	18.8
HF-DLPNO-CCSD(T_1)/CBS(DT)/CPS(56)	high level (model system)	0.6	–10.3
HF/def2-TZVPP	low level (real system)	27.9	21.3
HF-DLPNO-CCSD(T_1)/CBS(DT)/CPS(56)	high level (real system)	5.8	–7.3
ONIOM(DLPNO-CCSD(T_1) : HF)	(model sytem : real system)	5.7	–7.8

6.4 Thermochemistry of the full molecular system

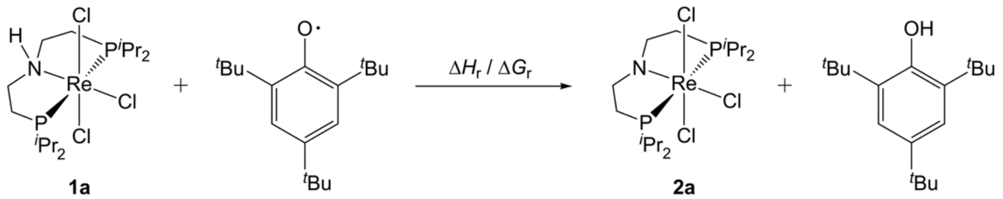

Table S13. Comparison of spin state energies, reaction energy and N–H/O–H dissociation energies calculated with methods. All energies in kcal mol^{-1}.

	ΔE_{S-T} (**1a**)	ΔE_{D-Q} (**2a**)	ΔE_r	ΔE_{N-H} (**1a**)	ΔE_{O-H} (Mes*OH)
ONIOM(HF-CCSD(T*)-F12/CBS(DT): HF-DLPNO-CCSD(T_1)/CBS(DT)/CPS(56))	4.2	–8.3	–18.1	69.3	87.4
HF-DLPNO-CCSD(T_1)/CBS(DT)/CPS(56)	5.8	–7.3	–17.7	71.6	89.3
PBE0-D3BJ/def2-TZVP	9.3	–3.7	–11.6	70.3	81.9
BP86-D3BJ/def2-TZVP	5.4	–9.9	–17.5	64.9	82.4

Table S14. Calculated thermochemical contributions (from PBE0-D3BJ/def2-TZVP Hessian analyses) and SOC contributions (from CASSCF/NEVPT2/QDPT calculations) to the reaction energy and the N–H/O–H dissociation energies. All energies in kcal mol^{-1}.

	$\Delta\Delta E_r$	$\Delta\Delta E_{N-H}$ (**1a**)	$\Delta\Delta E_{O-H}$ (Mes*OH)
$\Delta H_{contr.}$	–0.3	–	–
$\Delta G_{contr.}$	–0.4	–15.0	–15.8
ΔSOC_{QDPT}	6.2	6.2	–

Table S15. Comparison of computed reaction enthalpies and free reaction energies with experimental data. The superscript SOC denotes inclusion of spin orbit effects. All energies in kcal mol^{-1}.

	ΔH_r	$\Delta H_\mathrm{r}^\mathrm{SOC}$	ΔG_r	$\Delta G_\mathrm{r}^\mathrm{SOC}$
Experiment		–12.2 ± 0.1 [a]		–13.9 ± 2.6 [b]
ONIOM(HF-CCSD(T*)-F12/CBS(DT): HF-DLPNO-CCSD(T_1)/CBS(DT)/CPS(56))	–18.4	–12.2	–18.5	–12.3
HF-DLPNO-CCSD(T_1)/CBS(DT)/CPS(56)	–18.0	–11.8	–18.1	–11.9
PBE0-D3BJ/def2-TZVP	–11.9	–5.7	–12.0	–5.8
BP86-D3BJ/def2-TZVP	–17.8	–11.6	–17.9	–11.7

[a] in DCM [b] in THF

Table S16. Comparison of Computed $\mathrm{BDFE_{NH}}$ and $\mathrm{BDFE_{OH}}$ of **1a** and Mes*OH with experimental data. SOC denotes the inclusion of spin orbit effects. All energies in kcal mol^{-1}.

Method	$\mathrm{BDFE_{NH}}$ (**1a**)	$\mathrm{BDFE_{NH}^{SOC}}$ (**1a**)	$\mathrm{BDFE_{OH}}$(Mes*OH)
Experiment		60.5 ± 2.6 [a]	74.4 ± 1[b]
ONIOM(HF-CCSD(T*)-F12/CBS(DT): HF-DLPNO-CCSD(T_1)/CBS(DT)/CPS(56))	54.3	60.5	71.6
HF-DLPNO-CCSD(T_1)/CBS(DT)/CPS(56)	56.6	62.8	73.5
PBE0-D3BJ/def2-TZVP	55.3	61.5	66.1
BP86-D3BJ/def2-TZVP	49.8	56.0	66.6

[a] in DCM [b] in THF

6.5 State-Averaged CASSCF/NEVPT2/QDPT Computations

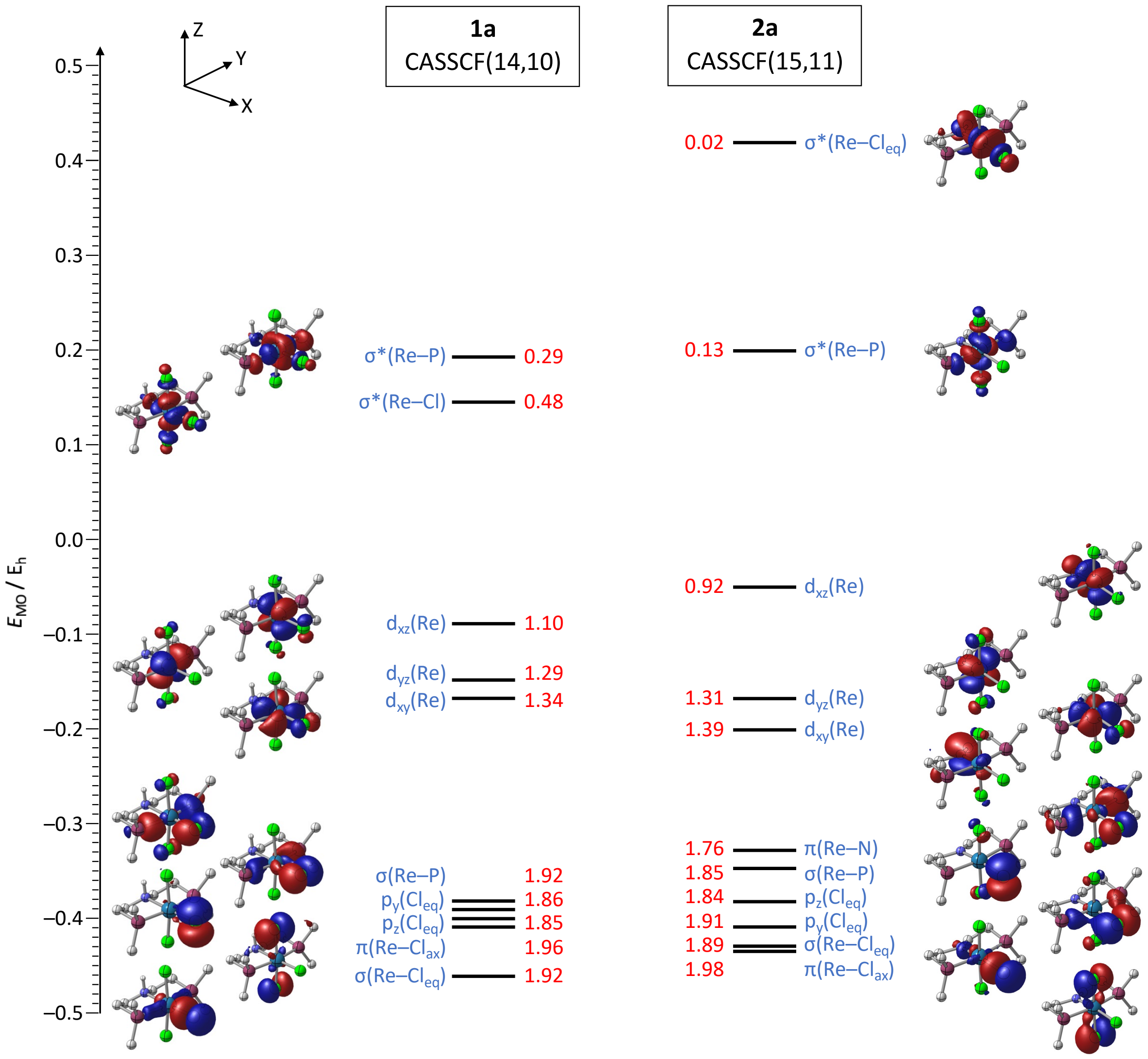

Figure S32. MO diagram, computed for **1a** in a CASSCF(14,10) expansion, state averaged over 50 singlet, 45 triplet and 5 quintet roots, and for **2a** in a CASSCF(15,11) expansion, state averaged over 40 doublet and 10 quartet roots. Selected hydrogen atoms and ^{i}Pr-groups not shown for clarity. Orbital isosurfaces at ± 0.05 $a_0^{-3/2}$.

Table S17. CASSCF(14,10)/NEVPT2 state compositions of **1a** with threshold for weights at 0.05 and cut-off for states at 10000 cm^{-1}.

State	Mult	E / cm^{-1}	Weight	Composition
0	3	0	0.50<br>0.46	2222212100<br>2222221100
1	3	124	0.50<br>0.46	2222221100<br>2222212100
2	1	1796	0.85	2222222000
3	1	5526	0.52<br>0.43	2222212100<br>2222221100
4	1	5892	0.51<br>0.43	2222221100<br>2222212100
5	3	7051	0.97	2222211200

Table S18. QDPT Eigenvectors of **1a** with threshold for weights at 0.05.

State	E / cm^{-1}	Weight	Block	Root	Spin	m_s
0	0	0.17<br>0.37<br>0.17<br>0.13	1<br>1<br>1<br>2	1<br>0<br>1<br>0	1<br>1<br>1<br>0	1<br>0<br>–1<br>0
1	1658	0.24<br>0.39<br>0.24	1<br>1<br>1	0<br>1<br>0	1<br>1<br>1	1<br>0<br>–1
2	2294	0.46<br>0.46	1<br>1	0<br>0	1<br>1	1<br>–1
3	2499	0.46<br>0.46	1<br>1	1<br>1	1<br>1	1<br>–1
4	3592	0.13<br>0.09<br>0.21<br>0.30<br>0.13<br>0.09	1<br>1<br>1<br>1<br>1<br>1	0<br>1<br>0<br>1<br>0<br>1	1<br>1<br>1<br>1<br>1<br>1	1<br>1<br>0<br>0<br>–1<br>–1
5	3645	0.09<br>0.14<br>0.27<br>0.23<br>0.09<br>0.14	1<br>1<br>1<br>1<br>1<br>1	0<br>1<br>0<br>1<br>0<br>1	1<br>1<br>1<br>1<br>1<br>1	1<br>1<br>0<br>0<br>–1<br>–1
6	5063	0.78	2	0	0	0

Table S19. CASSCF(15,11)/NEVPT2 state compositions of **2a** with threshold for weights at 0.05 and cut-off for states at 10000 cm^{-1}.

State	Mult	E / cm^{-1}	Weight	Composition
0	2	0	0.79<br>0.10	22222221000<br>22222212000
1	2	1511	0.80<br>0.10	22222212000<br>22222221000
2	4	5482	0.95	22222211100

Table S20. QDPT Eigenvectors of **2a** with threshold for weights at 0.05.

State	E / cm^{-1}	Weight	Block	Root	Spin	m_s
0	0	0.39<br>0.12<br>0.35<br>0.10	1<br>1<br>1<br>1	0<br>1<br>0<br>1	1/2<br>1/2<br>1/2<br>1/2	1/2<br>1/2<br>–1/2<br>–1/2
1	0	0.35<br>0.10<br>0.39<br>0.12	1<br>1<br>1<br>1	0<br>1<br>0<br>1	1/2<br>1/2<br>1/2<br>1/2	1/2<br>1/2<br>–1/2<br>–1/2
2	2422	0.08<br>0.33<br>0.11<br>0.36	1<br>1<br>1<br>1	0<br>1<br>0<br>1	1/2<br>1/2<br>1/2<br>1/2	1/2<br>1/2<br>–1/2<br>–1/2
3	2422	0.11<br>0.36<br>0.08<br>0.33	1<br>1<br>1<br>1	0<br>1<br>0<br>1	1/2<br>1/2<br>1/2<br>1/2	1/2<br>1/2<br>–1/2<br>–1/2
4	6541	0.35<br>0.18<br>0.15<br>0.27	0<br>0<br>0<br>0	0<br>0<br>0<br>0	3/2<br>3/2<br>3/2<br>3/2	3/2<br>1/2<br>–1/2<br>–3/2
5	6541	0.27<br>0.15<br>0.18<br>0.35	0<br>0<br>0<br>0	0<br>0<br>0<br>0	3/2<br>3/2<br>3/2<br>3/2	3/2<br>1/2<br>–1/2<br>–3/2
6	7015	0.50<br>0.08<br>0.29	0<br>0<br>0	0<br>0<br>0	3/2<br>3/2<br>3/2	1/2<br>–1/2<br>–3/2
7	7015	0.29<br>0.08<br>0.50	0<br>0<br>0	0<br>0<br>0	3/2<br>3/2<br>3/2	3/2<br>1/2<br>–1/2

Table S21. Influence of structural distortion on the energy separations of the two lowest spin-adapted states (triplet for **1a** and doublet for **2a**), ΔE_{NEVPT2}, and the SOC stabilization energies, ΔE_{SOC}. Molecular geometries were symmetrically distorted along both axial Re–Cl bonds by ±0.05 Å.

Re–Cl displacement / Å	Compound	ΔE_{NEVPT2} / kcal·mol^{-1}	ΔE_{SOC} / kcal·mol^{-1}	Compound	ΔE_{NEVPT2} / kcal·mol^{-1}	ΔE_{SOC} / kcal·mol^{-1}	$\Delta\Delta E_{SOC}$ / kcal·mol^{-1}
–0.05	**1a**	0.48	–9.71	**2a**	–6.81	–3.02	6.69
0	**1a**	0.36	–9.79	**2a**	–4.32	–3.61	6.18
+0.05	**1a**	1.46	–9.30	**2a**	–0.28	–5.09	4.21

6.6 Total energies of the H-truncated model system

Table S22. HF-CCSD(T*)-F12 total energies of the H-truncated model system.

HF-CCSD(T*)-F12	VDZ	VTZ	CBS(DT)
^{D}H	–0.499812	–0.499946	–0.499946
^{S}**1a**	–2354.084253	–2354.185199	–2354.217068
^{T}**1a**	–2354.081467	–2354.183069	–2354.215360
^{D}**2a**	–2353.473631	–2353.572496	–2353.603533
^{Q}**2a**	–2353.454891	–2353.554311	–2353.585645
^{S}Phenol	–307.024070	–307.061741	–307.071381
^{D}Phenoxy	–306.374111	–306.411067	–306.420534

Table S23. PBE0-CCSD(T*)-F12 total energies of the H-truncated model system.

PBE0-CCSD(T*)-F12	VDZ	VTZ	CBS(DT)
^{D}H	–0.499812	–0.499946	–0.499964
^{S}**1a**	–2354.065242	–2354.165366	–2354.198236
^{T}**1a**	–2354.062095	–2354.162993	–2354.196363
^{D}**2a**	–2353.454261	–2353.553500	–2353.585844
^{Q}**2a**	–2353.434954	–2353.535096	–2353.567839
^{S}Phenol	–307.019128	–307.057256	–307.067623
^{D}Phenoxy	–306.369423	–306.406716	–306.416869

Table S24. BP86-CCSD(T*)-F12 total energies of the H-truncated model system.

BP86-CCSD(T*)-F12	VDZ	VTZ	CBS(DT)
^{D}H	–0.499813	–0.499946	–0.499963
^{S}**1a**	–2354.045478	–2354.143845	–2354.177574
^{T}**1a**	–2354.042453	–2354.141677	–2354.175926
^{D}**2a**	–2353.434211	–2353.532292	–2353.565048
^{Q}**2a**	–2353.415100	–2353.514117	–2353.548016
^{S}Phenol	–307.015804	–307.053355	–307.063987
^{D}Phenoxy	–306.366093	–306.402815	–306.413227

Table S25. Extrapolated HF-DLPNO-CCSD(T_1) total energies of the H-truncated model system.

	CBS(DT)		CBS(TQ)	
	CPS(56)	CPS(67)	CPS(56)	CPS(67)
^{D}H	–0.499830	–0.499830	–0.500007	–0.500007
^{S}**1a**	–2354.229441	–2354.239220	–2354.241162	–2354.253295
^{T}**1a**	–2354.230371	–2354.239632	–2354.242507	–2354.253946
^{D}**2a**	–2353.614725	–2353.623787	–2353.629465	–2353.640512
^{Q}**2a**	–2353.598374	–2353.607833	–2353.615387	–2353.625419
^{S}Phenol	–307.088528	–307.089352	–307.078243	–307.079881
^{D}Phenoxy	–306.434474	–306.435953	–306.425677	–306.427374

Table S26. Extrapolated PBE0-DLPNO-CCSD(T_1) and BP86-DLPNO-CCSD(T_1) total energies of the H-truncated model system.

	PBE0-DLPNO-CCSD(T_1)		BP86-DLPNO-CCSD(T_1)	
	CBS(DT) CPS(56)	CBS(TQ) CPS(67)	CBS(DT) CPS(56)	CBS(TQ) CPS(67)
^{S}**1a**	–2354.224502	–2354.233697	–2354.214499	–2354.209064
^{T}**1a**	–2354.226046	–2354.233669	–2354.217526	–2354.209508
^{D}**2a**	–2353.610335	–2353.621502	–2353.601896	–2353.597070
^{Q}**2a**	–2353.595797	–2353.605707	–2353.588163	–2353.581670
^{S}Phenol	–307.088489	–307.074141	–307.089395	–307.068928
^{D}Phenoxy	–306.433203	–306.421648	–306.434611	–306.416597

Table S27. SCF total energies (E_{SCF}), reference total energies after the QRO transformation (E_{ref}) and DLPNO-CCSD(T1)/def2-TZVPP/T_{CutPNO} = 10^{-5} total energies ($E_{DLPNO-CCSD(T)}$) of **2a** in the H-truncated model system.

	^{2}**2a**			^{4}**2a**		
Reference orbitals	E_{SCF}	E_{ref}	$E_{DLPNO-CCSD(T1)}$	E_{SCF}	E_{ref}	$E_{DLPNO-CCSD(T1)}$
ROHF	–2350.677121	–2350.677105	–2353.165329	–2350.703433	–2350.703445	–2353.165380
UHF	–2350.685668	–2350.655316	–2353.167533	–2350.715594	–2350.703361	–2353.166299
ROKS (PBE0)	–2355.341962	–2350.576528	–2353.175898	–2355.328861	–2350.600397	–2353.173288
UKS(PBE0)	–2355.343514	–2350.576733	–2353.175882	–2355.333410	–2350.600301	–2353.173219
ROKS(BP86)	–2356.665177	–2350.485011	–2353.160654	–2356.644878	–2350.508317	–2353.158769
UKS(BP86)	–2356.666028	–2350.485680	–2353.160771	–2356.647612	–2350.509407	–2353.158647

Table S28. DFT and HF total energies of the H-truncated model system.

	def2-TZVP		def2-TZVPP
	PBE0-D3BJ	BP86-D3BJ	HF
^{D}H	–0.501036	–0.500024	–0.499809
^{S}**1a**	–2355.983429	–2357.327427	–2351.276148
^{T}**1a**	–2355.988952	–2357.326972	–2351.312399
^{D}**2a**	–2355.373971	–2356.721656	–2350.685668
^{Q}**2a**	–2355.363929	–2356.701685	–2350.715593
^{S}Phenol	–307.235852	–307.602152	–305.678192
^{D}Phenoxy	–306.590090	–306.956197	–305.084657

6.7 Total energies of the full molecular system

Table S29. Extrapolated HF-DLPNO-CCSD(T_1) total energies of the full molecular system.

	HF-DLPNO-CCSD(T_1)/CBS(DT)/CPS(56)
^{D}H	–0.500133
^{S}**1a**	–2825.421333
^{T}**1a**	–2825.430625
^{D}**2a**	–2824.816373
^{Q}**2a**	–2824.804743
^{S}Phenol	–778.229649
^{D}Phenoxy	–777.587228

Table S30. DFT and HF total energies of the full molecular system.

	def2-TZVP		def2-TZVPP
	PBE0-D3BJ	BP86-D3BJ	HF
^{D}H	–0.501036	–0.500024	–0.499809
^{S}**1a**	–2827.413110	–2829.352692	–2819.863791
^{T}**1a**	–2827.427856	–2829.361219	–2819.908226
^{D}**2a**	–2826.814730	–2828.757835	–2819.284944
^{Q}**2a**	–2826.808907	–2828.742095	–2819.318867
^{S}Phenol	–778.610535	–779.563488	–774.230521
^{D}Phenoxy	–777.978971	–778.932170	–773.648060

Table S31. ONIOM(HF-CCSD(T*)-F12/CBS(DT):HF-DLPNO-CCSD(T_1)/CBS(DT)/CPS(56)) total energies of the full molecular system.

	ONIOM(HF-CC-F12:HF-DLPNO-CC)
^{D}H	–0.499946
^{S}**1a**	–2825.408960
^{T}**1a**	–2825.415614
^{D}**2a**	–2824.805181
^{Q}**2a**	–2824.792014
^{S}Phenol	–778.212502
^{D}Phenoxy	–777.573289

7. References

1 a) APEX3 v2016.9-0 (SAINT/SADABS/SHELXT/SHELXL), Bruker AXS Inc., Madison, WI, USA, **2016**. b) Sheldrick, G. M. *Acta Cryst.* **2015**, A71, 3-8. c) Sheldrick, G M. *Acta Cryst*. **2015**, C71, 3-8. d) Sheldrick, G. M. *Acta Cryst*. **2008**, A64, 112-122.

2 Tshepelevitsh, S.; Kütt, A.; Lõkov, M.; Kaljurand, I.; Saame, J.; Heering, A.; Plieger, P. G.; Vianello, R.; Leito, I. *Eur. J. Org. Chem*. **2019**, 6735–6748.

3 Fuoss, R. M. *J. Am. Chem. Soc.* **1958**, *80*, 5059–5061.

4 Wise, C. F.; Agarwal, R. G.; Mayer, J. M. *J. Am. Chem. Soc.* **2020**, *142*, 10681–10691.

5 Schendzielorz, F.; Finger, M.; Abbenseth, J.; Würtele, C.; Krewald, V.; Schneider, S.; *Angew. Chem.* **2019**, 131, 840-844.
